# Proton pump inhibitors and dementia risk: Evidence from a cohort study using linked routinely collected national health data in Wales, UK

**DOI:** 10.1371/journal.pone.0237676

**Published:** 2020-09-18

**Authors:** Roxanne Cooksey, Jonathan Kennedy, Michael S. Dennis, Valentina Escott-Price, Ronan A. Lyons, Michael Seaborne, Sinead Brophy

**Affiliations:** 1 Health Data Research UK, Data Science, Swansea University Medical School, Swansea, Wales, United Kingdom; 2 National Centre for Population Health and Wellbeing Research, United Kingdom; 3 Cardiff University, Dementia Research Institute, School of Medicine, Cardiff, Wales, United Kingdom; University of Oxford, UNITED KINGDOM

## Abstract

**Objectives:**

Proton pump inhibitors (PPIs) are commonly prescribed for prevention and treatment of gastrointestinal conditions or for gastroprotection from other drugs. Research suggests they are linked to increased dementia risk. We use linked national health data to examine the association between PPI use and the development of incident dementia.

**Methods and findings:**

A population-based study using electronic health-data from the Secure Anonymised Information Linkage (SAIL) Databank, Wales (UK) from 1999 to 2015. Of data available on 3,765,744 individuals, a cohort who had ever been prescribed a PPI was developed (n = 183,968) for people aged 55 years and over and compared to non-PPI exposed individuals (131,110). Those with prior dementia, mild-cognitive-impairment or delirium codes were excluded. Confounding factors included comorbidities and/or drugs associated with them. Comorbidities might include head injury and some examples of medications include antidepressants, antiplatelets and anticoagulants. These commonly prescribed drugs were investigated as it was not feasible to explore all drugs in this study. The main outcome was a diagnosis of incident dementia. Cox proportional hazard regression modelling was used to calculate the Hazard ratio (HR) of developing dementia in PPI-exposed compared to unexposed individuals while controlling for potential confounders.

The mean age of the PPI exposed individuals was 69.9 years and 39.8% male while the mean age of the unexposed individuals was 72.1 years and 41.1% male. The rate of PPI usage was 58.4% (183,968) and incident dementia rate was 11.8% (37,148/315,078). PPI use was associated with decreased dementia risk (HR: 0.67, 95% CI: 0.65 to 0.67, p<0.01).

**Conclusions:**

This study, using large-scale, multi-centre health-data was unable to confirm an association between PPI use and increased dementia risk. Previously reported links may be associated with confounders of people using PPI’s, such as increased risk of cardiovascular disease and/or depression and their associated medications which may be responsible for any increased risk of developing dementia.

## Introduction

Proton pump inhibitors (PPIs) have revolutionised the management of stomach acid suppression, successfully managing patients with gastro-oesophageal reflux disease (GORD). They effectively resolve GORD symptoms in 60–80% of patients within 1–2 months [1] and their use is on the rise [2, 3]. Long-term PPI use may also be a prophylactic measure and serve as a gastro-protectant for concomitant medications such as aspirin, well known to irritate the digestive tract [4, 5].

However, evidence suggests that PPIs are overprescribed [6] and inappropriately prescribed [7, 8]. With increased prescription, particularly through Europe [9]; their cost to the National Health Service in the UK is more than £100 million each year [10].

Some of the safety concerns associated with long-term use of PPIs include: cancer [11, 12] masking symptoms of other underlying conditions [13], infections [14–18], celiac disease [19], vitamin-B_12_ deficiency [20], abnormal kidney function [21, 22], and more recently, dementia [23, 24]. The association between PPI and dementia is potentially mediated by a PPI-induced increase of the protein amyloid-beta that contributes to the pathophysiology of dementia [25, 26], or vitamin-B_12_ deficiency caused by malabsorption induced by PPIs [27].

It was recently reported that PPI medications were associated with an almost two-fold (44%) increased risk of developing dementia [23], causing wide-spread concern over their safety. The findings were highlighted by the media with little mention of methodological limitations. These limitations include: the study design with a selected population sourced from German health insurance data claims; a control group with significant differences from the PPI exposed group; and increased comorbidities. Comorbidities such as ischaemic heart disease and depression are risk factors for developing dementia. These could result in residual confounding, contributing to an increased incidence of dementia in PPI exposed individuals in previous studies [23]. A subsequent study, using German primary care data found that PPIs were actually associated with a decreased risk of dementia [28]. However, a meta-analysis published in 2016 showed an increase risk of dementia with PPI use, but only four observational studies could be included and heterogeneity and methodological shortcomings were highlighted [29].

Another study suggested that PPIs do not increase the risk of dementia or cognitive impairment and were associated with an approximate 20% risk reduction. This investigation relied on self-reporting of PPI use, which may result in misclassification and recall bias, especially if individuals had memory issues [30]. Other studies were also unable to find an association of PPI usage and cognitive impairment or dementia [31–34] including a case-control study which took the PPI duration of exposure in to account [35].

There are clearly challenges in evaluating the safety of PPIs [36] with studies reporting contradictory findings of an increased risk of dementia [23, 24, 29] or no associated risk [28, 30–32, 35].

We examine a vast source of linked, routinely collected health-data while correcting for known confounders to examine the relationship between PPI and dementia risk. We used a cohort study so that we could directly compare our findings to existing publications.

## Methods

### Routinely collected data

Data were obtained from the Secure Anonymised Information Linkage (SAIL) databank [37, 38] that allows the linkage of routine clinical records. These include primary care and mortality data collected by the Office of National Statistics (ONS). The SAIL databank houses multi-sourced records from 5 million of the living and deceased population of Wales. The records are anonymised using a split-file approach; the demographic and clinical data are divided and sent to a third party where a unique linking field is applied—removing any identifiers. This allows the files to be recombined later. We are then able to, retrospectively and prospectively, follow-up individuals anonymously through different data sources. Previous work using the SAIL databank to investigate PPIs has been published elsewhere [39].

Primary care data is captured using Read codes relating to diagnoses, medication and process-of-care codes [40]. The Office of National Statistics (ONS) Mortality dataset contains demographic, place and underlying cause of death data using the International Classification of Diseases (ICD) clinical coding system.

Data was included from December 1999 to September 2015 for optimum data coverage purposes at the time of analysis.

Individuals who had ever taken PPIs were those with Read code records for prescriptions of PPI medications. They were followed-up and compared to non-PPI exposed individuals. The individuals first PPI prescription date formed the index date for the PPI exposed group. A random index date within the follow-up period was used for the control population who did not receive a PPI. This avoided pre-selecting a period of known ill-health for controls. In addition, using a true event date could bias the control population to more active healthcare users, due to their visitation to the doctor and may be less generalisable as a result. Individuals of 55 years age or above were included due to the increased risk of gastrointestinal complaints with advancing age [41].

**Fig 1:**
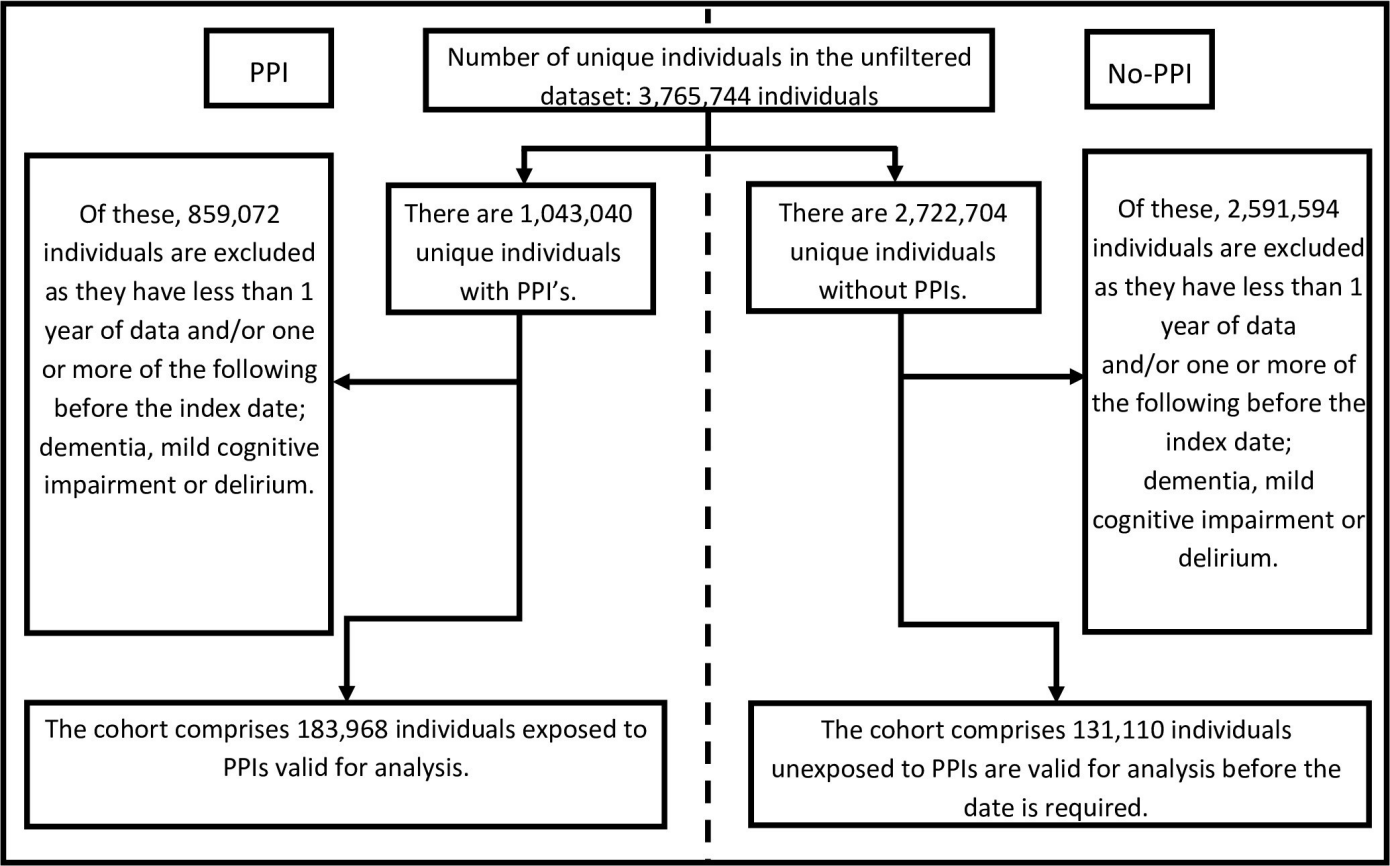
Cohort dataset creation diagram.

Of the 3,765,744 individuals in the SAIL databank who were aged 55 years or older, 1,043,040 had records of PPI use compared to 2,722,704 who did not. Individuals were excluded when they had less than one year of data available in their records from the index date and/or had records of dementia, mild cognitive impairment or delirium prior to the index date (Fig 1).

### Primary outcome

The primary outcome was dementia Read codes present in the primary care data post index date. The list of codes used for dementia classification is available in S1 Table of S1 File. These codes have been previously validated for use in UK epidemiological studies [42]. Data was linked to ONS data to obtain mortality data.

### Covariates of interest and confounding factors

The covariates were extracted at baseline from routine primary care data using the READ Code clinical coding system or derived data where appropriate. These included personal characteristics (age, sex, smoking status, obesity and alcohol consumption). The confounding comorbidities (diabetes, cardiovascular disease, depression, anxiety, head injury, hypertension, high cholesterol, vitamin-B_12_ deficiency and anaemia) and concomitant medications (anxiolytics, anti-depressants, anticoagulants, antiplatelets, statins, hormone replacement therapy (HRT), vitamin-B_12_ supplements, iron and antihypertensives) were extracted throughout the patient record; prior to or post- index date (S2 Table of S1 File). The rationale to include these confounders during the study period was to adjust for known factors that may occur in the long follow-up period. For instance, an extreme event such as a head injury or a long-term health condition, such as cardiovascular disease, which may occur in either PPI exposed or non-exposed group post-index date and these have been adjusted for in the model.

### Statistical analysis

Descriptive statistics were used to examine age, gender and covariate distribution for the participants of the cohort.

A step-wise cox proportional hazard regression model was employed to calculate hazard ratios while controlling for covariates and potential confounding factors. Univariate analysis (S3 Table of S1 File) determined factors to be included in the multivariate model. Censoring occurred when a patient died, was lost to follow-up (i.e. moved out of Wales resulting in incomplete health-records) or reaching the end of the study period (records up to 2015 at time of analysis).

Sensitivity analysis was performed, excluding patients who were taking vitamin-B12 which may help prevent cognitive decline [43].

Additionally, associations of developing dementia and the duration from first treatment with PPI were assessed in a sensitivity analysis comprising individuals who developed dementia within 2 to 4 years, 5 years to 9 years and 10 years post- index date compared to those who had not received PPIs.

Missing data were treated as missing throughout the study. All statistical analyses were conducted using STATA15.

### Ethical approval

Data held in the SAIL databank are anonymised and therefore, no ethical approval is required. All data contained in SAIL has the permission from the relevant Caldicott Guardian or Data Protection Officer. SAIL-related projects are required to obtain Information Governance Review Panel (IGRP) approval.

## Results

### Patient characteristics

The population consisted of 315,078 individuals, 40.3% were male (127184/315078) and the mean age was 70.8 years. The mean follow-up time for the whole cohort was 13.04 years (SD: 4.95). The proportion of PPI usage was 58.4% (183,968) and the incident dementia was 11.8% (37,148/315,078).

The PPI exposed group was slightly younger on average by 2.2 years. The proportion of females was slightly higher in the PPI exposed group (60.2%) compared to the unexposed group (58.9%) (Table 1).

**Table 1:** Characteristics of PPI exposed and unexposed patients at baseline.

	PPI		No PPI		Difference	
	n = 183,968		n = 131,110		(95% CI)	
Age^^^, mean years (SD)	69.9	(70)	72.1	(7.6)	2.2	(1.8 to 2.6)
Female, % (n)	60.2%	(110,720)	58.9%	(77,174)	1.3	(1.0 to 1.7)
BMI^^^, mean (SD)	26.3	(5.1)	25.8	(5.1)	0.5	(0.5 to 0.5)
Drink alcohol^^^, % (n)	3%	(5,510)	2.3%	(2,977)	0.7	(0.6 to 0.8)
Smoker^†^ % (n)	20.4%	(36,320)	29.9%	(37,290)	9.5	(9.2 to 9.8)
Dementia % (n)	11.4%	(21,023)	12.3%	(16,125)	0.9	(0.6 to 1.1)
Age at dementia diagnosis, mean years (SD)	82.1	(6.7)	81.5	(6.8)	0.6	(0.5 to 0.7)
Diabetes^†^ % (n)	19.4%	(35,778)	14.6%	(19,132)	4.8	(4.6 to 5.1)
Cardiovascular disease^†^ % (n)	53.9%	(99,065)	38%	(49,768)	15.9	(15.5 to 16.2)
Depression^†^ % (n)	32.7%	(60,167)	20.1%	(26,404)	12.6	(12.3 to 12.9)
Head injury^†^ % (n)	6.4%	(11,739)	4.8%	(6,315)	1.6	(1.4 to 1.7)
Vitamin B_12_ deficiency^†^ % (n)	0.8%	(1,516)	0.6%	(734)	0.2	(0.2 to 0.3)
Anaemia^†^ % (n)	19.1%	(35,153)	9.7%	(12,739)	9.4	(9.2 to 9.6)
Anxiolytics^†^ % (n)	29.1%	(53,434)	16.8%	(22,044)	12.3	(11.9 to 12.5)
Antidepressants^†^ % (n)	50.1%	(92,235)	28.9%	(37,847)	21.2	(20.9 to 21.6)
Anticoagulants^†^ % (n)	20.7%	(38,002)	14.7%	(19,257)	6	(5.7 to 6.2)
Antiplatelets^†^ % (n)	66.7%	(122,632)	47.2%	(61,838)	19.5	(19.2 to 19.8)
Statins ^†^% (n)	59.5%	(109,444)	36.9%	(48,343)	22.6	(22.3 to 23)
Hormone replacement therapy^†^ % (n)	9.9%	(18,274)	4.3%	(5,617)	5.6	(5.5 to 5.8)
Vitamin B_12_ medication^†^ % (n)	5.7%	(10,421)	3.4%	(4,506)	2.3	(2.1 to 2.4)
Anaemia medication^†^ % (n)	38.6%	(71,047)	19%	(24,933)	19.6	(19.3 to 19.9)
Antihypertensives^†^ % (n)	71.6%	(131,740)	54.1%	(70,925)	17.5	(17.2 to 17.9)
H_2_-receptor antagonists^†^ % (n)	19.1%	(35,203)	6.1%	(7,935)	13	(12.9 to 13)
Died % (n)	43.5%	(79,974)	53.3%	(69,917)	9.8	(9.5 to 10.2)
Mean age died, years (SD)	85	(6.0)	84.3	(5.8)	0.7	(0.6 to 0.8)

^^^ Covariates collected at index date.

^†^ Covariates collected throughout patient history, pre- or post- index date.

Missing data for PPI exposed: 36.7% for BMI (n = 67,569) and 3.3% (n = 6,057) for smoking status.

Missing data for non-exposed: 47.4% for BMI (n = 62,118) and 4.9% (n = 6,444) for smoking status.

Within the PPI exposed group, the incidence of dementia was 11.4% (21,023) and 12.3% (16,125) for the unexposed. The mean age at dementia diagnosis was similar in the PPI and non-PPI exposed groups; 82.1 years and 81.5 years, respectively (Table 1).

The mean follow-up time for PPI and non-PPI exposed was 10.9 years (SD: 4.0) and 8.6 years (SD: 4.4), respectively. The rate of death was significantly higher in the non-PPI (53.3%) exposed group compared to the PPI exposed group (43.5%) (Difference: 9.8, 95% CI: 9.5 to 10.2). The age of death was similar for both groups however the PPI group was slightly older (PPI: 85 years and non-PPI: 84.3 years) (Table 1).

The characteristics of the study populations are presented in Table 1 and S3 Table of S1 File.

### PPI and dementia

PPIs were not associated with an increased risk of dementia. Rather, PPI usage was associated with around a 30% reduced risk (Adjusted HR: 0.67, 95% CI: 0.65 to 0.69, p<0.001) (Tables 2 and 3); (Crude HR: 0.82, 95% CI: 0.80 to 0.84) (S3 Table of S1 File).

**Table 2:** Adjusted Hazard ratio of developing dementia when taking a PPI compared to a control population using a retrospective cohort.

	Hazard ratio	95% CI
PPI	0.67	0.65 to 0.69*
**Adjusted for:**		
Age	1.06	1.05 to 1.06*
Smoker	0.89	0.85 to 0.92*
BMI	0.95	0.95 to 0.95*
Diabetes	1.18	1.14 to 1.22*
Cardiovascular disease	0.93	0.90 to 0.97*
Depression	1.19	1.16 to 1.23*
Head injury	1.50	1.43 to 1.56*
Vitamin-B_12_ Deficiency	1.37	1.21 to 1.56*
Anxiolytics	1.62	1.57 to 1.67*
Antidepressants	1.66	1.61 to 1.72*
Anticoagulants	0.87	0.84 to 0.89*
Antiplatelets	1.72	1.66 to 1.79*
Statins	0.86	0.85 to 0.94*
Hormone replacement therapy	0.89	0.85 to 0.94*
Vitamin-B_12_ Medication	1.41	1.34 to 1.48*
Anaemia Medication	1.10	1.06 to 1.13*
Antihypertensives	0.71	0.69 to 0.74*
Histamine Receptor-^2^ Medication	0.89	0.85 to 0.94*

*P < 0.05.

*Gender*, *Anaemia and alcohol use were included in the model*, *but removed as did not reach* statistical significance.

**Table 3:** Sensitivity analysis: Adjusted hazard ratios of developing dementia for varying time points following PPI Index date compared to unexposed controls.

	Whole cohort							
	Including all dementia cases (n = 37,148)		Including dementia cases (n = 13,484) diagnosed 2 to 5 years post-index date		Including dementia cases (n = 9,822) diagnosed 5–9 years post-index date		Including dementia cases (n = 4,594) diagnosed 10 years or more post-index date	
			36% of all dementia cases		26.4% of all dementia cases		12.4% of all dementia cases	
	HR	95% CI	HR	95% CI	HR	95% CI	HR	95% CI
PPI	0.67	0.65 to 0.69*	0.58	0.56 to 0.60*	0.46	0.46 to 0.48*	0.29	0.28 to 0.30*
Age	1.05	1.05 to 1.06*	1.05	1.05 to 1.06*	1.06	1.05 to 1.06*	1.06	1.06 to 1.06*
Drink alcohol	1.10	1.01 to 1.18*	1.20	1.13 to 1.28*	1.18	1.10 to 1.25*	1.15	1.07 to 1.24*
Smoker	0.89	0.85 to 0.92*	0.89	0.86 to 0.92*	0.90	0.87 to 0.94*	0.91	0.87 to 0.95*
BMI	0.95	0.95 to 0.95*	0.95	0.95 to 0.95*	0.95	0.95 to 0.95*	0.95	0.95 to 0.95*
Diabetes	1.18	1.14 to 1.22*	1.18	1.14 to 1.23*	1.18	1.14 to 1.22*	1.18	1.13 to 1.23*
Cardiovascular disease	0.93	0.90 to 0.97*	0.94	0.91 to 0.98*	0.95	0.92 to 0.98*	0.95	0.92 to 0.99*
Depression	1.19	1.16 to 1.23*	1.19	1.15 to 1.24*	1.20	1.15 to 1.24*	1.17	1.12 to 1.21*
Head injury	1.49	1.43 to 1.56*	1.50	1.43 to 1.57*	1.51	1.43 to 1.59*	1.53	1.44 to 1.62*
Vitamin-B_12_ Deficiency	1.37	1.21 to 1.56*	1.42	1.25 to 1.61*	1.41	1.23 to 1.62*	1.44	1.23 to 1.69*
Anxiolytics	1.62	1.57 to 1.67*	1.65	1.60 to 1.70*	1.69	1.63 to 1.74*	1.77	1.70 to 1.84*
Antidepressants	1.66	1.61 to 1.72*	1.68	1.62 to 1.74*	1.68	1.61 to 1.74*	1.70	1.63 to 1.78*
Anticoagulants	0.87	0.84 to 0.91*	0.88	0.85 to 0.92*	0.88	0.84 to 0.92*	0.89	0.84 to 0.93*
Antiplatelets	1.72	1.66 to 1.79*	1.72	1.66 to 1.79*	1.73	1.66 to 1.80*	1.70	1.63 to 1.78*
Statins	0.86	0.83 to 0.89*	0.86	0.83 to 0.94*	0.87	0.83 to 0.90*	0.85	0.82 to 0.89*
Hormone replacement therapy	0.89	0.83 to 0.89*	0.89	0.84 to 0.94*	0.91	0.86 to 0.96*	0.96	0.90 to 1.02
Vitamin-B_12_ Medication	1.37	1.34 to 1.48*	1.44	1.37 to 1.52*	1.44	1.36 to 1.53*	1.46	1.36 to 1.56*
Anaemia Medication	1.09	1.06 to 1.13*	1.10	1.07 to 1.14*	1.11	1.07 to 1.15*	1.12	1.07 to 1.16*
Antihypertensives	0.71	0.69 to 0.73*	0.71	0.69 to 0.74*	0.71	0.68 to 0.73*	0.69	0.66 to 0.72*
Histamine Receptor-^2^ Medication	0.91	0.88 to 0.95*	0.97	0.93 to 1.01	1.04	1.0 to 1.09*	1.01	1.03 to 1.06*

*P < 0.05.

9,248 (24.9%) dementia cases were removed from this analysis as they developed dementia within two years of the PPI date. Gender, Anaemia and alcohol use were included in the model, but removed as did not reach statistical significance.

### Sensitivity analysis

A sensitivity analysis of removing vitamin B-12 deficiency from the model did not alter the results for the cohort study (S4 Table of S1 File).

To test the assumption that PPI initiation may predispose individuals to dementia, in a sensitivity analysis we investigated subgroups of individuals who were diagnosed with dementia following 2 to 4 years, 5 to 9 years and 10 years or more of first mention of PPI treatment. This demonstrated that when excluding individuals with varying exposures to PPIs, the hazard ratio of developing dementia reduced in the PPI exposed patients compared to the non-exposed and furthermore, as the time period increased (Table 3).

## Discussion

This study does not find evidence that PPI use is associated with an increased risk of dementia. Rather, in support for Booker et al., and Goldstein et al., our study, using large-scale, multi-sourced health-data, found that PPI usage was associated with a 30% lower risk of dementia. Our findings originate from data collected in a real-world setting which provided a heterogeneous sample, thereby overcoming selection bias which may have been an issue in previous studies.

The PPI exposed and non-exposed groups were slightly different. Those who were not treated with PPIs tended to be slightly older, smoked more and had less comorbidities and concomitant medications recorded in their routine health records compared to the PPI exposed group. There was also higher rate of death in the non-PPI exposed group compared to the PPI exposed. This may suggest that patients who are treated with PPIs are more closely monitored and are higher healthcare resource users; whereas patients who do not receive PPIs may visit their GP less overall.

When adjusted for covariates, some were associated with an increased risk of dementia (e.g. increasing age, head injury and depression). Other factors were associated with a reduced risk of dementia, including a lower BMI and cardiovascular disease. However, cardiovascular disease is an established risk factor for developing dementia [44]. Our findings may be explained by the increased risk of death in those who have serious comorbidities, meaning that they are at greater risk of dying earlier, before getting dementia.

However, some findings were interesting, for instance anticoagulants were associated with a lower risk of dementia in this study, while those taking antiplatelets developed dementia much earlier. However, this must be explored in additional study since this study was designed to explore risk of dementia with PPI usage.

It is important to consider that it is not known how long dementia takes to develop and how long the latent stages may last. Dementia may also not be diagnosed until later stages in the disease or on the other hand, may remain undiagnosed [45–47].

Therefore, by conducting sensitivity analysis omitting patients who were recently diagnosed with dementia following PPI treatment we could help to address the effects of misclassification bias. For instance, those who were diagnosed with dementia within one year of PPI treatment initiation would have been experiencing the early symptoms of dementia for some time already. These were existing dementia cases who would have been administered PPIs during the prodromal stage and so were misclassified. Patients who were diagnosed with dementia at 5 years and 10 years post-treatment initiation intervals also demonstrated an association of reduced incidence in dementia adding further support to the notion that PPIs are not associated with developing dementia. However, competing interest of death may also be an implication, which is often an issue with ageing cohorts. Nevertheless, no increased risk of dementia was associated with PPI usage in this study.

### Strengths

To our knowledge, this study involves the analysis of the largest-to-date follow-up of PPI and dementia cases from routinely collected health-records with up to 16 years average follow-up time. This allowed the assessment in a real-life setting which helps avoid selection bias and improve generalisability. Using routinely collected health-records for research purposes means that events are captured as they occur in the patient record rather than self-reported. Therefore, co-conditions, medications and head injuries for instance are confirmed in the data which overcomes recall bias in self-reported studies.

The use of a cohort study is also advantageous over a case-control design when using retrospective database studies, where all exposure and covariate data is already available. A case-control approach may result in inappropriate comparisons between exposure groups and lead to confounding [48]. Dementia can be a very slow developing condition however the long follow-up in this study meant that prodromal dementia could be followed until diagnosis. Dementia is also a complex, multifactorial condition and known potential confounders have been included in our analysis to help to reduce residual confounding, however this can never be assumed to be complete.

### Weaknesses

While routinely collected health-records are advantageous in their use for secondary research purposes, they may also have their disadvantages, such as missing data. This study does not take in to account the duration or dose of PPI treatment, however this has been investigated elsewhere and long-term use was not associated with an increased risk of developing AD [35]. Also, some PPIs are also available to purchase over-the-counter and so, we are unable to control for this within this study. Furthermore, a prescription for PPIs in the primary healthcare record does not necessarily guarantee that the individual has taken the medication which can lead to misclassification bias.

Pre-symptomatic dementia cases may also have been misclassified to the control population however this may be addressed by the time-lag sensitivity analysis.

We did not stratify on dementia type so that our findings were comparable to previous studies [23, 24, 28]. However, our future work will explore risk factors of developing dementia by dementia sub-type.

Given the advancing age of the cohort, the competing risk of death [49] is likely to be high which was not controlled for in this study. The rate of death was higher in the PPI unexposed group, however the age of death was similar for both groups and was beyond the mean age of dementia diagnosis. A weakness of the cox proportional hazard is that the risk of disease may be overestimated by failing to control for competing risk of death [49, 50]. As such, the hazard ratio may be slightly over-estimated, which in this instance, adds further support to the lack of association of PPI treatment with dementia in this cohort.

Finally, the rate of alcohol use was considerably low in the routine data. This may suggest that the rate of reporting alcohol was indeed low, had not been recorded well in primary care or be associated with the fact that the cohort was older and rate of drinking alcohol was truly low.

To conclude, our evidence suggests that PPIs are not associated with an increased risk of dementia. Rather, there is a 30% reduced risk associated with the widely prescribed gastro-protectants. Mechanisms behind potential protective effects of PPIs include anti-neurotoxic effects, such as reduction of brain oxidative stress and reduced neutrophil infiltration or inflammation in the brain [51, 52].

Further research in to the relationship between PPIs and a reduced risk of dementia, such as stratifying on the type of dementia can help us understand more about PPIs and their interactions during the pathogenesis of distinct dementias. Medications associated with a lower risk of dementia can be explored further, with regard to dose and duration of treatment for different dementias. Enhanced understanding of the associations between different medications and dementia not only supports greater understanding of the disease process itself, but can also earmark existing therapies to be investigated further as potential agents to be repurposed to target dementia.

This study finds no evidence for concern about developing dementia after taking a PPI. This may reassure people who are currently taking them, however due to the limitations of this study further research is needed, for instance, to ascertain the effect of duration of PPI exposure.

## Supporting information

S1 File (DOCX)
