# Supplementary material for: Proton pump inhibitors and dementia risk: Evidence from a cohort study using linked routinely collected national health data in Wales, UK
Source: PLoS One. 2020 Sep 18;15(9):e0237676. doi: 10.1371/journal.pone.0237676 (PMC7500586; doi:10.1371/journal.pone.0237676)
Supplement: S1 File — (DOCX) [file pone.0237676.s001.docx]

**Supplementary Table Index**

Supplementary Table 1: Dementia Read codes

Supplementary Table 2: Read codes used excluding dementia codes

Supplementary Table 3: Univariate/candidate variables to be included in model

Supplementary Table 4: Hazard ratio of developing dementia when taking a PPI compared to a control population using a retrospective cohort while omitting vitamin-B_12_ deficiency for sensitivity analysis.

**S1 Table: Dementia Read codes**

| **READ_CD** | **DESCRIPTION** |
| --- | --- |
| 1461 | H/O: dementia |
| 3AE.. | Global deterioration scale: assessment of prim deg dementia |
| 3AE1. | GDS level 2 – very mild cognitive decline |
| 3AE2. | GDS level 3 – mild cognitive decline |
| 3AE3. | GDS level 4 – moderate cognitive decline |
| 3AE4. | GDS level 5 – moderately severe cognitive decline |
| 3AE5. | GDS level 6 – severe cognitive decline |
| 3AE6. | GDS level 7 – very severe cognitive decline |
| 66h.. | Dementia monitoring |
| 6AB.. | Dementia annual review |
| 8Hla. | Referral to dementia care advisor |
| 9Ou1. | Dementia monitoring first letter |
| 9Ou2. | Dementia monitoring second letter |
| 9Ou3. | Dementia monitoring third letter |
| 9Ou4. | Dementia monitoring verbal invite |
| 9Ou5. | Dementia monitoring telephone invite |
| 9hD.. | Exception reporting: dementia quality indicators |
| 9hD0. | Excepted from dementia quality indicators: Patient unsuitabl |
| 9hD1. | Excepted from dementia quality indicators: Informed dissent |
| C251. | Wernicke’s encephalopathy |
| C253. | Wernicke’s encephalopathy |
| E00.. | Senile dementia |
| E00.. | Senile/presenile dementia |
| E000. | Uncomplicated senile dementia |
| E001. | Presenile dementia |
| E0010 | Uncomplicated presenile dementia |
| E0011 | Presenile dementia with delirium |
| E0012 | Presenile dementia with paranoia |
| E0013 | Presenile dementia with depression |
| E001z | Presenile dementia NOS |
| E002. | Senile dementia with depressive or paranoid features |
| E0020 | Senile dementia with paranoia |
| E0021 | Senile dementia with depression |
| E002z | Senile dementia with depressive or paranoid features NOS |
| E003. | Senile dementia with delirium |
| E004. | Arteriosclerotic dementia |
| E004. | Multi infarct dementia |
| E0040 | Uncomplicated arteriosclerotic dementia |
| E0041 | Arteriosclerotic dementia with delirium |
| E0042 | Arteriosclerotic dementia with paranoia |
| E0043 | Arteriosclerotic dementia with depression |
| E004z | Arteriosclerotic dementia NOS |
| E0110 | Korsakov’s alcoholic psychosis |
| **READ_CD** | **DESCRIPTION** |
| E0111 | Korsakov’s alcoholic psychosis with peripheral neuritis |
| E0112 | Wernicke-Korsakov syndrome |
| E012. | Other alcoholic dementia |
| E012. | Alcoholic dementia NOS |
| E0120 | Chronic alcoholic brain syndrome |
| E040. | Korsakoff's non-alcoholic psychosis |
| E041. | Dementia in conditions EC |
| Eu00. | [X]Dementia in Alzheimer's disease |
| Eu000 | [X]Dementia in Alzheimer's disease with early onset |
| Eu000 | [X]Presenile dementia,Alzheimer's type |
| Eu000 | [X]Primary degen dementia, Alzheimer's type, presenile onset |
| Eu000 | [X]Alzheimer's disease type 2 |
| Eu001 | [X]Dementia in Alzheimer's disease with late onset |
| Eu001 | [X]Alzheimer's disease type 1 |
| Eu001 | [X]Senile dementia,Alzheimer's type |
| Eu001 | [X]Primary degen dementia of Alzheimer's type, senile onset |
| Eu002 | [X]Dementia in Alzheimer's dis, atypical or mixed type |
| Eu00z | [X]Dementia in Alzheimer's disease, unspecified |
| Eu00z | [X]Alzheimer's dementia unspec |
| Eu01. | [X]Vascular dementia |
| Eu01. | [X]Arteriosclerotic dementia |
| Eu010 | [X]Vascular dementia of acute onset |
| Eu011 | [X]Multi-infarct dementia |
| Eu011 | [X]Predominantly cortical dementia |
| Eu012 | [X]Subcortical vascular dementia |
| Eu013 | [X]Mixed cortical and subcortical vascular dementia |
| Eu01y | [X]Other vascular dementia |
| Eu01z | [X]Vascular dementia, unspecified |
| Eu02. | [X]Dementia in other diseases classified elsewhere |
| Eu020 | [X]Dementia in Pick's disease |
| Eu021 | [X]Dementia in Creutzfeldt-Jakob disease |
| Eu022 | [X]Dementia in Huntington's disease |
| Eu023 | [X]Dementia in Parkinson's disease |
| Eu024 | [X]Dementia in human immunodef virus [HIV] disease |
| Eu025 | [X]Lewy body dementia |
| Eu02y | [X]Dementia in other specified diseases classif elsewhere |
| Eu02z | [X] Unspecified dementia |
| Eu02z | [X] Presenile dementia NOS |
| Eu02z | [X] Primary degenerative dementia NOS |
| Eu02z | [X] Senile dementia NOS |
| Eu02z | [X] Senile dementia, depressed or paranoid type |
| Eu03. | [X]Korsakov's psychosis, nonalcoholic |
| Eu041 | [X]Delirium superimposed on dementia |
| Eu106 | [X]Korsakov's psychosis, alcohol induced |
| Eu107 | [X]Alcoholic dementia NOS |
| **READ_CD** | **DESCRIPTION** |
| Eu107 | [X]Chronic alcoholic brain syndrome |
| F110. | Alzheimer's disease |
| F1100 | Alzheimer's disease with early onset |
| F1101 | Alzheimer's disease with late onset |
| F111. | Pick's disease |
| F112. | Senile degeneration of brain |
| F116. | Lewy body disease |
| ZS78D | Wernicke's dysphasia |
| ZS78D | Wernicke's aphasia |

**S2 Table: Read codes used excluding dementia codes**

| **READ_CD** | | **DESCRIPTION** | |  |
| --- | --- | --- | --- | --- |
| **CARDIOVASCULAR DISEASE** | | | |  |
| 14A3. | | H/O: myocardial infarct <60 | |  |
| 14A4. | | H/O: myocardial infarct >60 | |  |
| 14A5. | | H/O: angina pectoris | |  |
| 14A6. | | H/O: heart failure | |  |
| 14AH. | | H/O: Myocardial infarction in last year | |  |
| 14AJ. | | H/O: Angina in last year | |  |
| 14AM. | | H/O: Heart failure in last year | |  |
| 14AN. | | H/O: atrial fibrillation | |  |
| 14AR. | | History of atrial flutter | |  |
| 14AT. | | History of myocardial infarction | |  |
| 14AW. | | H/O acute coronary syndrome | |  |
| 14NB. | | H/O: Peripheral vascular disease procedure | |  |
| 1J60. | | Suspected heart failure | |  |
| 1O1.. | | Heart failure confirmed | |  |
| 21264 | | Heart failure resolved | |  |
| 323.. | | ECG: myocardial infarction | |  |
| 3232 | | ECG: old myocardial infarction | |  |
| 323Z. | | ECG: myocardial infarct NOS | |  |
| 3272 | | ECG: atrial fibrillation | |  |
| 3273 | | ECG: atrial flutter | |  |
| 388D. | | New York Heart Assoc classification heart failure symptoms | |  |
| 661M5 | | Heart failure self-management plan agreed | |  |
| 662p. | | Heart failure 6 month review | |  |
| 662S. | | Atrial fibrillation monitoring | |  |
| 662T. | | Congestive heart failure monitoring | |  |
| 662W. | | Heart failure annual review | |  |
| 679W1 | | Education about deteriorating heart failure | |  |
| 679X. | | Heart failure education | |  |
| 67D4. | | Heart failure information given to patient | |  |
| 68B6. | | Heart failure screen | |  |
| 6A9.. | | Atrial fibrillation annual review | |  |
| **READ_CD** | | **DESCRIPTION** | |  |
| 790D7 | | Replacement of valved cardiac conduit | |  |
| 7910 | | Plastic repair of mitral valve | |  |
| 79100 | | Allograft replacement of mitral valve | |  |
| 79101 | | Xenograft replacement of mitral valve | |  |
| 79102 | | Prosthetic replacement of mitral valve | |  |
| 79103 | | Replacement of mitral valve NEC | |  |
| 79104 | | Mitral valvuloplasty NEC | |  |
| 7910y | | Other specified plastic repair of mitral valve | |  |
| 7910z | | Plastic repair of mitral valve NOS | |  |
| 7911 | | Plastic repair of aortic valve | |  |
| 79110 | | Allograft replacement of aortic valve | |  |
| 79111 | | Xenograft replacement of aortic valve | |  |
| 79112 | | Prosthetic replacement of aortic valve | |  |
| 79113 | | Replacement of aortic valve NEC | |  |
| 79114 | | Aortic valvuloplasty NEC | |  |
| 79115 | | Transapical aortic valve implantation | |  |
| 79116 | | Transluminal aortic valve implantation | |  |
| 7911y | | Other specified plastic repair of aortic valve | |  |
| 7911z | | Plastic repair of aortic valve NOS | |  |
| 7914 | | Plastic repair of unspecified valve of heart | |  |
| 79140 | | Allograft replacement of valve of heart NEC | |  |
| 79141 | | Xenograft replacement of valve of heart NEC | |  |
| 79142 | | Prosthetic replacement of valve of heart NEC | |  |
| 79143 | | Replacement of valve of heart NEC | |  |
| 79146 | | Replacement of truncal valve | |  |
| 79150 | | Revision of plastic repair of mitral valve | |  |
| 79151 | | Revision of plastic repair of aortic valve | |  |
| 79160 | | Open mitral valvotomy | |  |
| 79170 | | Closed mitral valvotomy | |  |
| 79180 | | Annuloplasty of mitral valve | |  |
| 79190 | | Percutaneous transluminal mitral valvotomy | |  |
| 792.. | | Coronary artery operations | |  |
| 7920 | | Saphenous vein graft replacement of coronary artery | |  |
| 7921 | | Other autograft replacement of coronary artery | |  |
| 7922 | | Allograft replacement of coronary artery | |  |
| 7923 | | Prosthetic replacement of coronary artery | |  |
| 7924 | | Revision of bypass for coronary artery | |  |
| 79240 | | Revision of bypass for one coronary artery | |  |
| 79241 | | Revision of bypass for two coronary arteries | |  |
| 79242 | | Revision of bypass for three coronary arteries | |  |
| 79243 | | Revision of bypass for four or more coronary arteries | |  |
| 7924y | | Other specified revision of bypass for coronary artery | |  |
| 7924z | | Revision of bypass for coronary artery NOS | |  |
| 7925 | | Connection of mammary artery to coronary artery | |  |
| 79275 | | Open angioplasty of coronary artery | |  |
| **READ_CD** | | **DESCRIPTION** | |  |
| 7928 | | Transluminal balloon angioplasty of coronary artery | |  |
| 79280 | | Percut transluminal balloon angioplasty one coronary artery | |  |
| 79281 | | Percut translum balloon angioplasty mult coronary arteries | |  |
| 79282 | | Percut translum balloon angioplasty bypass graft coronary a | |  |
| 79283 | | Percut translum cutting balloon angioplasty coronary artery | |  |
| 7928y | | Transluminal balloon angioplasty of coronary artery OS | |  |
| 7928z | | Transluminal balloon angioplasty of coronary artery NOS | |  |
| 79290 | | Percutaneous transluminal laser coronary angioplasty | |  |
| 79293 | | Rotary blade coronary angioplasty | |  |
| 79294 | | Insertion of coronary artery stent | |  |
| 79295 | | Insertion of drug-eluting coronary artery stent | |  |
| 792D. | | Other bypass of coronary artery | |  |
| 792Dy | | Other specified other bypass of coronary artery | |  |
| 792Dz | | Other bypass of coronary artery NOS | |  |
| 7936A | | Implant intravenous pacemaker for atrial fibrillation | |  |
| 793G. | | Perc translumin balloon angioplasty stenting coronary artery | |  |
| 793Gy | | OS perc translumina balloon angioplast stenting coronary art | |  |
| 793Gz | | Perc translum balloon angioplasty stenting coronary art NOS | |  |
| 793M1 | | Perc transluminal ablation of atrial wall for atrial flutter | |  |
| 793M2 | | Percutaneous transluminal internal cardioversion NEC | |  |
| 793M3 | | Perc translum ablat conduct sys heart for atrial flutter NEC | |  |
| 7L1H. | | External resuscitation | |  |
| 7L1H0 | | Direct current cardioversion | |  |
| 7L1H1 | | External cardioversion NEC | |  |
| 7L1H2 | | Internal electrode cardioversion | |  |
| 7L1H8 | | Chemical cardioversion | |  |
| 840.. | | Direct current cardioversion planned | |  |
| 889A. | | Diab mellit insulin-glucose infus acute myocardial infarct | |  |
| 8CeC. | | Preferred place of care for next exacerbation heart failure | |  |
| 8CL3. | | Heart failure care plan discussed with patient | |  |
| 8CMK. | | Has heart failure management plan | |  |
| 8CMW2 | | Atrial fibrillation care pathway | |  |
| 8CMW8 | | Heart failure clinical pathway | |  |
| 8H2S. | | Admit heart failure emergency | |  |
| 8H44. | | Cardiological referral | |  |
| 8H440 | | Referral to cardiology multidisciplinary team | |  |
| 8H5G. | | Referral to Cardiothoracic surgeon | |  |
| 8H7v. | | Referral to cardiac rehabilitation nurse | |  |
| 8HBE. | | Heart failure follow-up | |  |
| 8HBJ. | | Stroke / transient ischaemic attack referral | |  |
| 8Hg8. | | Discharge from practice nurse heart failure clinic | |  |
| 8HgD. | | Discharge from heart failure nurse service | |  |
| 8HHb. | | Referral to heart failure nurse | |  |
| 8HHM. | | Ref to multidisciplinary stroke function improvement service | |  |
| 8HHW. | | Referral for warfarin monitoring | |  |
| **READ_CD** | | **DESCRIPTION** | |  |
| 8HHz. | | Referral to heart failure exercise programme | |  |
| 8Hk0. | | Referred to heart failure education group | |  |
| 8Hkk. | | Referral to cardiac rehabilitation programme | |  |
| 8Hkl. | | Referral to cardiac rehabilitation service by secondary care | |  |
| 8Hkt. | | Referral to community cardiology service | |  |
| 8HQ7. | | Referral for echocardiography | |  |
| 8HR9. | | Referral for 24 hour ECG | |  |
| 8HRA. | | Referral for exercise ECG | |  |
| 8HRD. | | Referral for ambulatory electrocardiogram | |  |
| 8HRF. | | Referral for cardiac pacemaker check | |  |
| 8HRG. | | Referral for cardiac event recording | |  |
| 8HTL. | | Referral to heart failure clinic | |  |
| 8HTL0 | | Referral to rapid access heart failure clinic | |  |
| 8HTQ. | | Referral to stroke clinic | |  |
| 8HTs. | | Referral to community anticoagulation clinic | |  |
| 8HTy. | | Referral to atrial fibrillation clinic | |  |
| 8HVE. | | Private referral cardiothoracic surgeon | |  |
| 8HVJ. | | Private referral to cardiologist | |  |
| 8IB8. | | Referral to heart failure exercise programme not indicated | |  |
| 8IE1. | | Referral to heart failure exercise programme declined | |  |
| 8L40. | | Coronary artery bypass graft operation planned | |  |
| 8L41. | | Coronary angioplasty planned | |  |
| 8OAD. | | Provision of written information about atrial fibrillation | |  |
| 9hF.. | | Exception reporting: atrial fibrillation quality indicators | |  |
| 9hF1. | | Excepted from atrial fibrillation qual indic: Inform dissent | |  |
| 9hH.. | | Exception reporting: heart failure quality indicators | |  |
| 9hH0. | | Excepted heart failure quality indicators: Patient unsuitabl | |  |
| 9hH1. | | Excepted heart failure quality indicators: Informed dissent | |  |
| 9hS.. | | Exception report: peripherl arterial disease quality indicat | |  |
| 9m5.. | | High risk of heart failure screening invitation | |  |
| 9N0k. | | Seen in heart failure clinic | |  |
| 9N2p. | | Seen by community heart failure nurse | |  |
| 9N4s. | | Did not attend practice nurse heart failure clinic | |  |
| 9N4w. | | Did not attend heart failure clinic | |  |
| 9N6T. | | Referred by heart failure nurse specialist | |  |
| 9Or.. | | Heart failure monitoring administration | |  |
| 9Or0. | | Heart failure review completed | |  |
| 9Or1. | | Heart failure monitoring telephone invite | |  |
| 9Or2. | | Heart failure monitoring verbal invite | |  |
| 9Or3. | | Heart failure monitoring first letter | |  |
| 9Or4. | | Heart failure monitoring second letter | |  |
| 9Or5. | | Heart failure monitoring third letter | |  |
| 9Os.. | | Atrial fibrillation monitoring administration | |  |
| 9Os0. | | Atrial fibrillation monitoring first letter | |  |
| 9Os1. | | Atrial fibrillation monitoring second letter | |  |
| **READ_CD** | | **DESCRIPTION** | |  |
| 9Os2. | | Atrial fibrillation monitoring third letter | |  |
| 9Os3. | | Atrial fibrillation monitoring verbal invite | |  |
| 9Os4. | | Atrial fibrillation monitoring telephone invite | |  |
| G11.. | | Mitral valve diseases | |  |
| G110. | | Mitral stenosis | |  |
| G112. | | Mitral stenosis with insufficiency | |  |
| G113. | | Nonrheumatic mitral valve stenosis | |  |
| G114. | | Ruptured mitral valve cusp | |  |
| G11z. | | Mitral valve disease NOS | |  |
| G12z. | | Rheumatic aortic valve disease NOS | |  |
| G13.. | | Diseases of mitral and aortic valves | |  |
| G130. | | Mitral and aortic stenosis | |  |
| G131. | | Mitral stenosis and aortic insufficiency | |  |
| G132. | | Mitral insufficiency and aortic stenosis | |  |
| G133. | | Mitral and aortic incompetence | |  |
| G13y. | | Multiple mitral and aortic valve involvement | |  |
| G13z. | | Mitral and aortic valve disease NOS | |  |
| G1yz1 | | Rheumatic left ventricular failure | |  |
| G232. | | Hypertensive heart&renal dis wth (congestive) heart failure | |  |
| G3... | | Ischaemic heart disease | |  |
| G30.. | | Acute myocardial infarction | |  |
| G301. | | Other specified anterior myocardial infarction | |  |
| G301z | | Anterior myocardial infarction NOS | |  |
| G304. | | Posterior myocardial infarction NOS | |  |
| G305. | | Lateral myocardial infarction NOS | |  |
| G306. | | True posterior myocardial infarction | |  |
| G3071 | | Acute non-ST segment elevation myocardial infarction | |  |
| G308. | | Inferior myocardial infarction NOS | |  |
| G30B. | | Acute posterolateral myocardial infarction | |  |
| G30X. | | Acute transmural myocardial infarction of unspecif site | |  |
| G30X0 | | Acute ST segment elevation myocardial infarction | |  |
| G30y. | | Other acute myocardial infarction | |  |
| G30yz | | Other acute myocardial infarction NOS | |  |
| G30z. | | Acute myocardial infarction NOS | |  |
| G31.. | | Other acute and subacute ischaemic heart disease | |  |
| G310. | | Postmyocardial infarction syndrome | |  |
| G311. | | Preinfarction syndrome | |  |
| G3110 | | Myocardial infarction aborted | |  |
| G3111 | | Unstable angina | |  |
| G3113 | | Refractory angina | |  |
| G3115 | | Acute coronary syndrome | |  |
| G31y. | | Other acute and subacute ischaemic heart disease | |  |
| G31y0 | | Acute coronary insufficiency | |  |
| G31yz | | Other acute and subacute ischaemic heart disease NOS | |  |
| G32.. | | Old myocardial infarction | |  |
| **READ_CD** | | **DESCRIPTION** | |  |
| G33.. | | Angina pectoris | |  |
| G331. | | Prinzmetal's angina | |  |
| G33z. | | Angina pectoris NOS | |  |
| G33z3 | | Angina on effort | |  |
| G33z5 | | Post infarct angina | |  |
| G33z7 | | Stable angina | |  |
| G33zz | | Angina pectoris NOS | |  |
| G34.. | | Other chronic ischaemic heart disease | |  |
| G340. | | Coronary atherosclerosis | |  |
| G34y. | | Other specified chronic ischaemic heart disease | |  |
| G34yz | | Other specified chronic ischaemic heart disease NOS | |  |
| G34z. | | Other chronic ischaemic heart disease NOS | |  |
| G35.. | | Subsequent myocardial infarction | |  |
| G350. | | Subsequent myocardial infarction of anterior wall | |  |
| G351. | | Subsequent myocardial infarction of inferior wall | |  |
| G353. | | Subsequent myocardial infarction of other sites | |  |
| G35X. | | Subsequent myocardial infarction of unspecified site | |  |
| G36.. | | Certain current complication follow acute myocardial infarct | |  |
| G38.. | | Postoperative myocardial infarction | |  |
| G380. | | Postoperative transmural myocardial infarction anterior wall | |  |
| G381. | | Postoperative transmural myocardial infarction inferior wall | |  |
| G383. | | Postoperative transmural myocardial infarction unspec site | |  |
| G384. | | Postoperative subendocardial myocardial infarction | |  |
| G38z. | | Postoperative myocardial infarction, unspecified | |  |
| G3y.. | | Other specified ischaemic heart disease | |  |
| G3z.. | | Ischaemic heart disease NOS | |  |
| G540. | | Mitral valve incompetence | |  |
| G5400 | | Mitral incompetence, non-rheumatic | |  |
| G5401 | | Mitral incompetence, cause unspecified | |  |
| G5402 | | Mitral valve prolapse | |  |
| G5403 | | Mitral valve leaf prolapse | |  |
| G540z | | Mitral valve disorders NOS | |  |
| G541. | | Aortic valve disorders | |  |
| G5414 | | Aortic valve stenosis with insufficiency | |  |
| G541z | | Aortic valve disorders NOS | |  |
| G544. | | Multiple valve diseases | |  |
| G5441 | | Disorders of both mitral and tricuspid valves | |  |
| G5442 | | Combined disorders of mitral, aortic and tricuspid valves | |  |
| G544X | | Multiple valve disease, unspecified | |  |
| G573. | | Atrial fibrillation and flutter | |  |
| G5730 | | Atrial fibrillation | |  |
| G5731 | | Atrial flutter | |  |
| G5732 | | Paroxysmal atrial fibrillation | |  |
| G5733 | | Non-rheumatic atrial fibrillation | |  |
| G5734 | | Permanent atrial fibrillation | |  |
| **READ_CD** | | **DESCRIPTION** | |  |
| G5735 | | Persistent atrial fibrillation | |  |
| G5736 | | Paroxysmal atrial flutter | |  |
| G573z | | Atrial fibrillation and flutter NOS | |  |
| G58.. | | Heart failure | |  |
| G580. | | Congestive heart failure | |  |
| G5800 | | Acute congestive heart failure | |  |
| G5801 | | Chronic congestive heart failure | |  |
| G5804 | | Congestive heart failure due to valvular disease | |  |
| G581. | | Left ventricular failure | |  |
| G5810 | | Acute left ventricular failure | |  |
| G582. | | Acute heart failure | |  |
| G583. | | Heart failure with normal ejection fraction | |  |
| G584. | | Right ventricular failure | |  |
| G58z. | | Heart failure NOS | |  |
| G5y4z | | Post cardiac operation heart failure NOS | |  |
| G670. | | Cerebral atherosclerosis | |  |
| G677. | | Occlusion/stenosis cerebral arts not result cerebral infarct | |  |
| G7... | | Arterial, arteriole and capillary disease | |  |
| G70.. | | Atherosclerosis | |  |
| G700. | | Aortic atherosclerosis | |  |
| G701. | | Renal artery atherosclerosis | |  |
| G702. | | Extremity artery atheroma | |  |
| G702z | | Extremity artery atheroma NOS | |  |
| G70y. | | Other specified artery atheroma | |  |
| G70y0 | | Carotid artery atherosclerosis | |  |
| G73.. | | Other peripheral vascular disease | |  |
| G734. | | Peripheral arterial disease | |  |
| G73y. | | Other specified peripheral vascular disease | |  |
| G73yz | | Other specified peripheral vascular disease NOS | |  |
| G73z. | | Peripheral vascular disease NOS | |  |
| G73z0 | | Intermittent claudication | |  |
| G73z1 | | Spasm of peripheral artery | |  |
| G73zz | | Peripheral vascular disease NOS | |  |
| G7y.. | | Other specified arterial, arteriole or capillary disease | |  |
| G7z.. | | Arterial, arteriole and capillary diseases NOS | |  |
| Gyu10 | | [X]Other mitral valve diseases | |  |
| Gyu3. | | [X]Ischaemic heart diseases | |  |
| Gyu30 | | [X]Other forms of angina pectoris | |  |
| Gyu32 | | [X]Other forms of acute ischaemic heart disease | |  |
| Gyu33 | | [X]Other forms of chronic ischaemic heart disease | |  |
| Gyu34 | | [X]Acute transmural myocardial infarction of unspecif site | |  |
| Gyu36 | | [X]Subsequent myocardial infarction of unspecified site | |  |
| Gyu55 | | [X]Other nonrheumatic mitral valve disorders | |  |
| Gyu56 | | [X]Other aortic valve disorders | |  |
| Gyu5A | | [X]Aortic valve disorders in diseases classified elsewhere | |  |
| **READ_CD** | | **DESCRIPTION** | |  |
| Gyu5D | | [X]Multiple valve disorders/diseases CE | |  |
| Gyu70 | | [X]Atherosclerosis of other arteries | |  |
| Gyu74 | | [X]Other specified peripheral vascular diseases | |  |
| P641. | | Bicuspid aortic valve | |  |
| P65.. | | Congenital mitral stenosis | |  |
| P650. | | Congenital mitral stenosis, unspecified | |  |
| P652. | | Parachute deformity of the mitral valve | |  |
| P65z. | | Congenital mitral stenosis NOS | |  |
| P66.. | | Congenital mitral insufficiency | |  |
| SP002 | | Mechanical complication of heart valve prosthesis | |  |
| SP003 | | Mechanical complication of coronary bypass | |  |
| SP076 | | Coronary artery bypass graft occlusion | |  |
| SP084 | | Heart transplant failure and rejection | |  |
| SP085 | | Heart-lung transplant failure and rejection | |  |
| SP111 | | Cardiac insufficiency as a complication of care | |  |
| SyuK6 | | [X]Oth complics of cardiac & vasc prosth devices/impl/graft | |  |
| TB012 | | Implant of heart valve prosthesis + complication, no blame | |  |
| ZV433 | | [V]Has artificial heart valve | |  |
| ZV457 | | [V]Presence of aortocoronary bypass graft | |  |
| ZV458 | | [V]Presence of coronary angioplasty implant and graft | |  |
| ZV45H | | [V]Presence of prosthetic heart valve | |  |
| ZV45K | | [V]Presence of coronary artery bypass graft | |  |
| ZV45L | | [V]Status following coronary angioplasty NOS | |  |
| ZVu6e | | [X]Presence of other heart valve replacement | |  |
| **DIABETES** | | | |  |
| C1001 | | Diabetes mellitus, adult onset, no mention of complication | |  |
| C1011 | | Diabetes mellitus, adult onset, with ketoacidosis | |  |
| C1021 | | Diabetes mellitus, adult onset, with hyperosmolar coma | |  |
| C1031 | | Diabetes mellitus, adult onset, with ketoacidotic coma | |  |
| C1041 | | Diabetes mellitus, adult onset, with renal manifestation | |  |
| C1051 | | Diabetes mellitus, adult onset, + ophthalmic manifestation | |  |
| C1061 | | Diabetes mellitus, adult onset, + neurological manifestation | |  |
| C1071 | | Diabetes mellitus, adult, + peripheral circulatory disorder | |  |
| C1072 | | Diabetes mellitus, adult with gangrene | |  |
| C1074 | | NIDDM with peripheral circulatory disorder | |  |
| C109. | | Non-insulin dependent diabetes mellitus | |  |
| C1090 | | Non-insulin-dependent diabetes mellitus with renal comps | |  |
| C1091 | | Non-insulin-dependent diabetes mellitus with ophthalm comps | |  |
| C1092 | | Non-insulin-dependent diabetes mellitus with neuro comps | |  |
| C1093 | | Non-insulin-dependent diabetes mellitus with multiple comps | |  |
| C1094 | | Non-insulin dependent diabetes mellitus with ulcer | |  |
| C1095 | | Non-insulin dependent diabetes mellitus with gangrene | |  |
| C1096 | | Non-insulin-dependent diabetes mellitus with retinopathy | |  |
| C1097 | | Non-insulin dependent diabetes mellitus - poor control | |  |
| C1099 | | Non-insulin-dependent diabetes mellitus without complication | |  |
| **READ_CD** | | **DESCRIPTION** | |  |
| C109A | | Non-insulin dependent diabetes mellitus with mononeuropathy | |  |
| C109B | | Non-insulin dependent diabetes mellitus with polyneuropathy | |  |
| C109C | | Non-insulin dependent diabetes mellitus with nephropathy | |  |
| C109D | | Non-insulin dependent diabetes mellitus with hypoglyca coma | |  |
| C109E | | Non-insulin depend diabetes mellitus with diabetic cataract | |  |
| C109F | | Non-insulin-dependent d m with peripheral angiopath | |  |
| C109G | | Non-insulin dependent diabetes mellitus with arthropathy | |  |
| C109H | | Non-insulin dependent d m with neuropathic arthropathy | |  |
| C109J | | Insulin treated Type 2 diabetes mellitus | |  |
| C109K | | Hyperosmolar non-ketotic state in type 2 diabetes mellitus | |  |
| C10F. | | Type 2 diabetes mellitus | |  |
| C10F0 | | Type 2 diabetes mellitus with renal complications | |  |
| C10F1 | | Type 2 diabetes mellitus with ophthalmic complications | |  |
| C10F2 | | Type 2 diabetes mellitus with neurological complications | |  |
| C10F3 | | Type 2 diabetes mellitus with multiple complications | |  |
| C10F4 | | Type 2 diabetes mellitus with ulcer | |  |
| C10F5 | | Type 2 diabetes mellitus with gangrene | |  |
| C10F6 | | Type 2 diabetes mellitus with retinopathy | |  |
| C10F7 | | Type 2 diabetes mellitus - poor control | |  |
| C10F9 | | Type 2 diabetes mellitus without complication | |  |
| C10FA | | Type 2 diabetes mellitus with mononeuropathy | |  |
| C10FB | | Type 2 diabetes mellitus with polyneuropathy | |  |
| C10FC | | Type 2 diabetes mellitus with nephropathy | |  |
| C10FD | | Type 2 diabetes mellitus with hypoglycaemic coma | |  |
| C10FE | | Type 2 diabetes mellitus with diabetic cataract | |  |
| C10FF | | Type 2 diabetes mellitus with peripheral angiopathy | |  |
| C10FG | | Type 2 diabetes mellitus with arthropathy | |  |
| C10FH | | Type 2 diabetes mellitus with neuropathic arthropathy | |  |
| C10FJ | | Insulin treated Type 2 diabetes mellitus | |  |
| C10FK | | Hyperosmolar non-ketotic state in type 2 diabetes mellitus | |  |
| C10FL | | Type 2 diabetes mellitus with persistent proteinuria | |  |
| C10FM | | Type 2 diabetes mellitus with persistent microalbuminuria | |  |
| C10FN | | Type 2 diabetes mellitus with ketoacidosis | |  |
| C10FQ | | Type 2 diabetes mellitus with exudative maculopathy | |  |
| C10FR | | Type 2 diabetes mellitus with gastroparesis | |  |
| C10y1 | | Diabetes mellitus, adult, + other speciﬁed manifestation | |  |
| C10z1 | | Diabetes mellitus, adult onset, + unspeciﬁed complication | |  |
| L1806 | | Pre-existing diabetes mellitus, non-insulin-dependent | |  |
| **DEPRESSION** | | | |  |
| E112. | Single major depressive episode | | |  |
| E1120 | Single major depressive episode, unspecified | | |  |
| E1121 | Single major depressive episode, mild | | |  |
| E1122 | Single major depressive episode, moderate | | |  |
| E1123 | Single major depressive episode, severe without psychosis | | |  |
| E1125 | Single major depressive episode, partial or unspec remission | | |  |
| E1126 | Single major depressive episode, in full remission | | |  |
| E112z | Single major depressive episode NOS | | |  |
| E113. | Recurrent major depressive episode | | |  |
| E1130 | Recurrent major depressive episodes, unspecified | | |  |
| E1131 | Recurrent major depressive episodes, mild | | |  |
| E1132 | Recurrent major depressive episodes, moderate | | |  |
| E1133 | Recurrent major depressive episodes, severe, no psychosis | | |  |
| E1135 | Recurrent major depressive episodes, partial/unspec remission | | |  |
| E1136 | Recurrent major depressive episodes, in full remission | | |  |
| E1137 | Recurrent depression | | |  |
| E113z | Recurrent major depressive episode NOS | | |  |
| E118. | Seasonal affective disorder | | |  |
| E135. | Agitated depression | | |  |
| E2003 | Anxiety with depression | | |  |
| E204. | Neurotic depression reactive type | | |  |
| E291. | Prolonged depressive reaction | | |  |
| E2B.. | Depressive disorder NEC | | |  |
| E2B0. | Postviral depression | | |  |
| E2B1. | Chronic depression | | |  |
| Eu32. | [X]Depressive episode | | |  |
| Eu320 | [X]Mild depressive episode | | |  |
| Eu321 | [X]Moderate depressive episode | | |  |
| Eu322 | [X]Severe depressive episode without psychotic symptoms | | |  |
| Eu324 | [X]Mild depression | | |  |
| Eu32y | [X]Other depressive episodes | | |  |
| Eu32z | [X]Depressive episode, unspecified | | |  |
| Eu33. | [X]Recurrent depressive disorder | | |  |
| Eu330 | [X]Recurrent depressive disorder, current episode mild | | |  |
| Eu331 | [X]Recurrent depressive disorder, current episode moderate | | |  |
| Eu332 | [X]Recurrent depressive disorder cur epi severe without psyc sympt | | |  |
| Eu334 | [X]Recurrent depressive disorder, currently in remission | | |  |
| Eu33y | [X]Other recurrent depressive disorders | | |  |
| Eu33z | [X]Recurrent depressive disorder, unspecified | | |  |
| Eu341 | [X]Dysthymia | | |  |
| Eu412 | [X]Mixed anxiety and depressive disorder | | |  |
| E200. | Anxiety states | | |  |
| E2000 | Anxiety state unspecified | | |  |
| E2001 | Panic disorder | | |  |
| E2002 | Generalised anxiety disorder | | |  |
| E2004 | Chronic anxiety | | |  |
| E2005 | Recurrent anxiety | | |  |
| E200z | Anxiety state NOS | | |  |
| Eu41. | [X]Other anxiety disorders | | |  |
| Eu410 | [X]Panic disorder [episodic paroxysmal anxiety] | | |  |
| Eu411 | [X]Generalised anxiety disorder | | |  |
| Eu413 | [X]Other mixed anxiety disorders | | |  |
| Eu41y | [X]Other specified anxiety disorders | | |  |
| Eu41z | [X]Anxiety disorder, unspecified | | |  |
| 1B12. | Nerves, nervousness | | |  |
| 1B13. | Anxiousness | | |  |
| 1B17. | Depressed | | |  |
| 1B1U. | Symptoms of depression | | |  |
| 1BQ.. | Loss of capacity for enjoyment | | |  |
| 1BT.. | Depressed mood | | |  |
| 1BU.. | Loss of hope for the future | | |  |
| 2257 | O/E – depressed | | |  |
| 2258 | O/E – anxious | | |  |
| 2259 | O/E – nervous | | |  |
| **HEAD INJURY** | | | |  |
| S0... | | Fracture of skull | |  |
| S00.. | | Fracture of vault of skull | |  |
| S000. | | Closed fracture vault of skull without intracranial injury | |  |
| S0000 | | Closed #skull vlt no intracranial injury, unspec state consc | |  |
| S0001 | | Closed #skull vlt no intracranial injury, no loss of consc | |  |
| S0002 | | Closed #skull vlt no intracranial injury, <1hr loss of consc | |  |
| S0003 | | Closed #skull vlt no intracranial injury, 1-24hr loss consc | |  |
| S0004 | | Closed #skull vlt no intracranial injury, >24hr LOC+recovery | |  |
| S0005 | | Closed #skull vlt no intracranial inj,>24hr LOC not restored | |  |
| S0006 | | Closed #skull vlt no intracranial inj, LOC unspec duration | |  |
| S000z | | Closed #skull vlt no intracranial injury + concussion unspec | |  |
| S001. | | Closed fracture vault of skull with intracranial injury | |  |
| S0010 | | Closed #skull vlt + intracranial injury, unspec state consc | |  |
| S0011 | | Closed #skull vlt + intracranial injury, no loss of consc | |  |
| S0012 | | Closed #skull vlt + intracranial injury, <1hr loss of consc | |  |
| S0013 | | Closed #skull vlt + intracranial injury, 1-24hr loss consc | |  |
| S0014 | | Closed #skull vlt + intracranial injury, >24hr LOC+recovery | |  |
| S0015 | | Closed #skull vlt + intracranial inj, >24hr LOC not restored | |  |
| S0016 | | Closed #skull vlt + intracranial injury, LOC unspec duration | |  |
| S001z | | Closed #skull vlt with intracranial injury+concussion unspec | |  |
| S002. | | Open fracture vault of skull without intracranial injury | |  |
| S0020 | | Open #skull vlt no intracranial injury, unspec state consc | |  |
| S0021 | | Open #skull vlt no intracranial injury, no loss of consc | |  |
| S0022 | | Open #skull vlt no intracranial injury, <1hr loss of consc | |  |
| S0023 | | Open #skull vlt no intracranial injury, 1-24hr loss of consc | |  |
| S0024 | | Open #skull vlt no intracranial injury, >24hr LOC+recovery | |  |
| S0025 | | Open #skull vlt no intracranial inj, >24hr LOC not restored | |  |
| S0026 | | Open #skull vlt no intracranial injury, LOC unspec duration | |  |
| S002z | | Open #skull vlt no intracranial injury + concussion unspec | |  |
| S003. | | Open fracture vault of skull with intracranial injury | |  |
| S0030 | | Open #skull vlt + intracranial injury, unspec state of consc | |  |
| S0031 | | Open #skull vlt + intracranial injury, no loss of consc | |  |
| S0032 | | Open #skull vlt + intracranial injury, <1hr loss of consc | |  |
| S0033 | | Open #skull vlt + intracranial injury, 1-24hr loss of consc | |  |
| S0034 | | Open #skull vlt + intracranial injury, >24hr LOC + recovery | |  |
| S0035 | | Open #skull vlt + intracranial inj, >24hr LOC not restored | |  |
| S0036 | | Open #skull vlt + intracranial injury, LOC unspec duration | |  |
| S003z | | Open #skull vlt with intracranial injury + concussion unspec | |  |
| S00z. | | Fracture of vault of skull NOS | |  |
| S01.. | | Fracture of base of skull | |  |
| S010. | | Closed fracture base of skull without intracranial injury | |  |
| S0100 | | Closed #skull bse no intracranial injury, unspec state consc | |  |
| S0101 | | Closed #skull bse no intracranial injury, no loss of consc | |  |
| S0102 | | Closed #skull bse no intracranial injury, <1hr loss of consc | |  |
| S0103 | | Closed #skull bse no intracranial injury, 1-24hr loss consc | |  |
| S0104 | | Closed #skull bse no intracranial injury, >24hr LOC+recovery | |  |
| S0105 | | Closed #skull bse no intracranial inj,>24hr LOC not restored | |  |
| S0106 | | Closed #skull bse no intracranial inj, LOC unspec duration | |  |
| S010z | | Closed #skull bse no intracranial injury + concussion unspec | |  |
| S011. | | Closed fracture base of skull with intracranial injury | |  |
| S0110 | | Closed #skull bse + intracranial inj, unspec state of consc | |  |
| S0111 | | Closed #skull bse + intracranial injury, no loss of consc | |  |
| S0112 | | Closed #skull bse + intracranial injury, <1hr loss of consc | |  |
| S0113 | | Closed #skull bse + intracranial injury, 1-24hr loss consc | |  |
| S0114 | | Closed #skull bse + intracranial injury, >24hr LOC+recovery | |  |
| S0115 | | Closed #skull bse + intracranial inj, >24hr LOC not restored | |  |
| S0116 | | Closed #skull bse + intracranial injury, LOC unspec duration | |  |
| S011z | | Closed #skull bse + intracranial injury + concussion unspec | |  |
| S012. | | Open fracture base skull without mention intracranial injury | |  |
| S0120 | | Open #skull bse no intracranial inj, unspec state of consc | |  |
| S0121 | | Open #skull bse no intracranial injury, no loss of consc | |  |
| S0122 | | Open #skull bse no intracranial injury, <1hr loss of consc | |  |
| S0123 | | Open #skull bse no intracranial injury, 1-24hr loss of consc | |  |
| S0124 | | Open #skull bse no intracranial injury, >24hr LOC+recovery | |  |
| S0125 | | Open #skull bse no intracranial inj, >24hr LOC not restored | |  |
| S0126 | | Open #skull bse no intracranial injury, LOC unspec duration | |  |
| S012z | | Open #skull bse no intracranial injury + concussion unspec | |  |
| S013. | | Open fracture base of skull with intracranial injury | |  |
| S0130 | | Open #skull bse + intracranial injury, unspec state of consc | |  |
| S0131 | | Open #skull bse + intracranial injury, no loss of consc | |  |
| S0132 | | Open #skull bse + intracranial injury, <1hr loss of consc | |  |
| S0133 | | Open #skull bse + intracranial injury, 1-24hr loss of consc | |  |
| S0134 | | Open #skull bse + intracranial injury, >24hr LOC + recovery | |  |
| S0135 | | Open #skull bse + intracranial inj, >24hr LOC not restored | |  |
| S0136 | | Open #skull bse + intracranial injury, LOC unspec duration | |  |
| S013z | | Open #skull bse + intracranial injury + concussion unspec | |  |
| S01z. | | Fracture of base of skull NOS | |  |
| S02.. | | Fracture of face bones | |  |
| S020. | | Closed fracture nose | |  |
| S021. | | Open fracture nose | |  |
| S022. | | Fracture of mandible, closed | |  |
| S0220 | | Closed fracture mandible (site unspecified) | |  |
| S0221 | | Closed fracture of mandible, condylar process | |  |
| S0222 | | Closed fracture of mandible, subcondylar | |  |
| S0223 | | Closed fracture of mandible, coronoid process | |  |
| S0224 | | Closed fracture of mandible, ramus, unspecified | |  |
| S0225 | | Closed fracture of mandible, angle of jaw | |  |
| S0226 | | Closed fracture of mandible, symphysis of body | |  |
| S0227 | | Closed fracture of mandible, alveolar border of body | |  |
| S0228 | | Closed fracture of mandible, body, other and unspecified | |  |
| S022x | | Closed fracture of mandible, multiple sites | |  |
| S022z | | Fracture of mandible, closed, NOS | |  |
| S023. | | Fracture of mandible, open | |  |
| S0230 | | Open fracture mandible (site unspecified) | |  |
| S0231 | | Open fracture of mandible, condylar process | |  |
| S0232 | | Open fracture of mandible, subcondylar | |  |
| S0233 | | Open fracture of mandible, coronoid process | |  |
| S0234 | | Open fracture of mandible, ramus, unspecified | |  |
| S0235 | | Open fracture of mandible, angle of jaw | |  |
| S0236 | | Open fracture of mandible, symphysis of body | |  |
| S0237 | | Open fracture of mandible, alveolar border of body | |  |
| S0238 | | Open fracture of mandible, body, other and unspecified | |  |
| S023x | | Open fracture of mandible, multiple sites | |  |
| S023z | | Fracture of mandible, open, NOS | |  |
| S024. | | Fracture of malar or maxillary bones, closed | |  |
| S0240 | | Closed fracture maxilla | |  |
| S0241 | | Closed fracture zygoma | |  |
| S024z | | Fracture of malar or maxillary bones, closed, NOS | |  |
| S025. | | Fracture of malar or maxillary bones, open | |  |
| S0250 | | Open fracture maxilla | |  |
| S0251 | | Open fracture zygoma | |  |
| S025z | | Fracture of malar or maxillary bones, open, NOS | |  |
| S026. | | Closed orbital blow-out fracture | |  |
| S027. | | Open orbital blow-out fracture | |  |
| S028. | | Fracture of skull and facial bones | |  |
| S0280 | | Fracture of nasal bones | |  |
| S0281 | | Fracture of orbital floor | |  |
| S0282 | | Fracture of malar and maxillary bones | |  |
| S0283 | | Fracture of mandible | |  |
| S02A. | | Le Fort I fracture maxilla | |  |
| S02B. | | Le Fort II fracture maxilla | |  |
| S02C. | | Le Fort III fracture maxilla | |  |
| S02x. | | Closed fracture other facial bone | |  |
| S02x0 | | Fracture of alveolus, closed | |  |
| S02x1 | | Fracture of orbit NOS, closed | |  |
| S02x2 | | Fracture of palate, closed | |  |
| S02xz | | Fracture of other facial bones, closed, NOS | |  |
| S02y. | | Open fracture other facial bone | |  |
| S02y0 | | Fracture of alveolus, open | |  |
| S02y1 | | Fracture of orbit NOS, open | |  |
| S02y2 | | Fracture of palate, open | |  |
| S02yz | | Fracture of other facial bones,open, NOS | |  |
| S02z. | | Fracture of facial bone NOS | |  |
| S03.. | | Other and unqualified skull fractures | |  |
| S030. | | Closed fracture of skull NOS without intracranial injury | |  |
| S0300 | | Closed #skull NOS no intracranial inj, unspec state of consc | |  |
| S0301 | | Closed #skull NOS no intracranial inj, no loss of consc | |  |
| S0302 | | Closed #skull NOS no intracranial inj, <1hr loss of consc | |  |
| S0303 | | Closed #skull NOS no intracranial inj, 1-24hr loss of consc | |  |
| S0304 | | Closed #skull NOS no intracranial inj, >24hrs LOC + recovery | |  |
| S0305 | | Closed #skull NOS no intracranial inj,>24hr LOC not restored | |  |
| S0306 | | Closed #skull NOS no intracranial inj, LOC unspec duration | |  |
| S030z | | Closed #skull NOS no intracranial inj + concussion unspec | |  |
| S031. | | Closed fracture of skull NOS with intracranial injury | |  |
| S0310 | | Closed #skull NOS + intracranial inj, unspec state of consc | |  |
| S0311 | | Closed #skull NOS + intracranial inj, no loss of consc | |  |
| S0312 | | Closed #skull NOS + intracranial inj, <1hr loss of consc | |  |
| S0313 | | Closed #skull NOS + intracranial inj, 1-24hrs loss of consc | |  |
| S0314 | | Closed #skull NOS + intracranial inj, >24hrs LOC + recovery | |  |
| S0315 | | Closed #skull NOS + intracranial inj, >24hr LOC not restored | |  |
| S0316 | | Closed #skull NOS + intracranial inj, LOC unspec duration | |  |
| S031z | | Closed #skull NOS + intracranial inj + concussion unspec | |  |
| S032. | | Open #skull NOS without mention of intracranial injury | |  |
| S0320 | | Open #skull NOS no intracranial inj, unspec state of consc | |  |
| S0321 | | Open #skull NOS no intracranial inj, no loss of consc | |  |
| S0322 | | Open #skull NOS no intracranial inj, <1hr loss of consc | |  |
| S0323 | | Open #skull NOS no intracranial inj, 1-24hrs loss of consc | |  |
| S0324 | | Open #skull NOS no intracranial inj, >24hrs LOC + recovery | |  |
| S0325 | | Open #skull NOS no intracranial inj, >24hrs LOC not restored | |  |
| S0326 | | Open #skull NOS no intracranial inj, LOC unspec duration | |  |
| S032z | | Open #skull NOS no intracranial inj + concussion unspec | |  |
| S033. | | Open fracture of skull NOS with intracranial injury | |  |
| S0330 | | Open #skull NOS + intracranial inj, unspec state of consc | |  |
| S0331 | | Open #skull NOS + intracranial inj, no loss of consc | |  |
| S0332 | | Open #skull NOS + intracranial inj, <1hr loss of consc | |  |
| S0333 | | Open #skull NOS + intracranial inj, 1-24hrs loss of consc | |  |
| S0334 | | Open #skull NOS + intracranial inj, >24hrs LOC + recovery | |  |
| S0335 | | Open #skull NOS + intracranial inj, >24hrs LOC not restored | |  |
| S0336 | | Open #skull NOS + intracranial inj, LOC unspec duration | |  |
| S033z | | Open #skull NOS + intracranial inj + concussion unspec | |  |
| S03z. | | Skull fracture NOS | |  |
| S04.. | | Multiple fractures involving skull or face with other bones | |  |
| S040. | | Mult #skull/face+other bones, closed, no intracranial injury | |  |
| S0400 | | Closed #skull/face, mult, no intracranial inj, unspec consc | |  |
| S0401 | | Closed #skull/face, mult, no intracranial inj, no loss consc | |  |
| S0402 | | Closed #skull/face, mult, no intracranial inj, <1hr LOC | |  |
| S0403 | | Closed #skull/face, mult, no intracranial inj, 1-24hrs LOC | |  |
| S0404 | | Closed #skull/face, mult,no intracran inj,>24hr LOC+recovery | |  |
| S0405 | | Closed #skull/face,mult,no intracran inj,>24hr LOC-restored | |  |
| S0406 | | Closed #skull/face,mult,no intracran inj,LOC unspec duration | |  |
| S040z | | Closed #skull/face,mult,no intracran inj, concussion unspec | |  |
| S041. | | Mult #skull/face+other bones, closed + intracranial injury | |  |
| S0410 | | Closed #skull/face, mult + intracranial inj, unspec consc | |  |
| S0411 | | Closed #skull/face, mult + intracranial inj, no loss consc | |  |
| S0412 | | Closed #skull/face, mult + intracranial inj, <1hr LOC | |  |
| S0413 | | Closed #skull/face, mult + intracranial inj, 1-24hrs LOC | |  |
| S0414 | | Closed #skull/face, mult+intracran inj, >24hr LOC+recovery | |  |
| S0415 | | Closed #skull/face, multi+intracran inj, >24hr LOC-restored | |  |
| S0416 | | Closed #skull/face,mult + intracran inj, LOC unspec duration | |  |
| S041z | | Closed #skull/face,mult + intracran inj, concussion unspec | |  |
| S042. | | Mult #skull/face + other bones, open, no intracranial injury | |  |
| S0420 | | Open #skull/face, mult, no intracranial inj, unspec consc | |  |
| S0421 | | Open #skull/face, mult, no intracranial inj, no loss consc | |  |
| S0422 | | Open #skull/face, mult, no intracranial inj, <1hr LOC | |  |
| S0423 | | Open #skull/face, mult, no intracranial inj, 1-24hrs LOC | |  |
| S0424 | | Open #skull/face, mult, no intracran inj, >24hr LOC+recovery | |  |
| S0425 | | Open #skull/face,mult,no intracran inj,>24hr LOC no restored | |  |
| S0426 | | Open #skull/face,mult,no intracran inj, LOC unspec duration | |  |
| S042z | | Open #skull/face,mult,no intracran inj, concussion unspec | |  |
| S043. | | Mult #skull/face + other bones, open + intracranial injury | |  |
| S0430 | | Open #skull/face, mult + intracranial inj, unspec consc | |  |
| S0431 | | Open #skull/face, mult + intracranial inj, no loss consc | |  |
| S0432 | | Open #skull/face, mult + intracranial inj, <1hr LOC | |  |
| S0433 | | Open #skull/face, mult + intracranial inj, 1-24hrs LOC | |  |
| S0434 | | Open #skull/face, mult + intracran inj, >24hr LOC + recovery | |  |
| S0435 | | Open #skull/face,mult + intracran inj, >24hr LOC no restored | |  |
| S0436 | | Open #skull/face, mult + intracran inj, LOC unspec duration | |  |
| S043z | | Open #skull/face, mult + intracran inj + concussion, unspec | |  |
| S044. | | Multiple fractures involving skull and facial bones | |  |
| S04z. | | Multiple fractures involving skull/face with other bones NOS | |  |
| S0z.. | | Fracture of skull NOS | |  |
| S6... | | Intracranial injury excluding those with skull fracture | |  |
| S60.. | | Concussion | |  |
| S600. | | Concussion with no loss of consciousness | |  |
| S601. | | Concussion with less than 1 hour loss of consciousness | |  |
| S602. | | Concussion with 1-24 hours loss of consciousness | |  |
| S603. | | Concussion with >24 hrs loss of consc with full recovery | |  |
| S604. | | Concussion with >24 hrs loss of consc without full recovery | |  |
| S605. | | Concussion with loss of consciousness of unspec duration | |  |
| S60z. | | Concussion NOS | |  |
| S61.. | | Cerebral laceration and contusion | |  |
| S610. | | Closed cerebral contusion | |  |
| S6100 | | Cortex cont no open intracranial wnd + unspec state consc | |  |
| S6101 | | Cortex cont no open intracranial wnd + no loss of consc | |  |
| S6102 | | Cortex cont no open intracranial wnd + <1hr loss of consc | |  |
| S6103 | | Cortex cont no open intracranial wnd + 1-24hrs loss of consc | |  |
| S6104 | | Cortex cont no open intracranial wnd + >24 hr LOC + recovery | |  |
| S6105 | | Cortex cont no open intracranial wnd +>24hr LOC not restored | |  |
| S6106 | | Cortex cont no open intracranial wnd + LOC unspec duration | |  |
| S610z | | Cortex cont no open intracranial wnd + concussion unspec | |  |
| S611. | | Open cerebral contusion | |  |
| S6110 | | Cortex cont + open intracranial wound + unspec state consc | |  |
| S6111 | | Cortex cont + open intracranial wound + no loss of consc | |  |
| S6112 | | Cortex cont + open intracranial wound + <1hr loss of consc | |  |
| S6113 | | Cortex cont + open intracranial wound + 1-24hr loss of consc | |  |
| S6114 | | Cortex cont + open intracranial wnd + >24hr LOC + recovery | |  |
| S6115 | | Cortex cont + open intracranial wnd + >24hr LOC not restored | |  |
| S6116 | | Cortex cont + open intracranial wnd + LOC unspec duration | |  |
| S611z | | Cortex cont + open intracranial wound + concussion unspec | |  |
| S612. | | Cortex laceration without mention of open intracranial wound | |  |
| S6120 | | Cortex lacn no open intracranial wound + unspec state consc | |  |
| S6121 | | Cortex lacn no open intracranial wound + no loss of consc | |  |
| S6122 | | Cortex lacn no open intracranial wound + <1hr loss of consc | |  |
| S6123 | | Cortex lacn no open intracranial wnd + 1-24hr loss of consc | |  |
| S6124 | | Cortex lacn no open intracranial wnd + >24hr LOC + recovery | |  |
| S6125 | | Cortex lacn no open intracranial wnd +>24hr LOC not restored | |  |
| S6126 | | Cortex lacn no open intracranial wnd + LOC unspec duration | |  |
| S612z | | Cortex lacn no open intracranial wound + concussion unspec | |  |
| S613. | | Cortex laceration with open intracranial wound | |  |
| S6130 | | Cortex lacn + open intracranial wound + unspec state consc | |  |
| S6131 | | Cortex lacn + open intracranial wound + no loss of consc | |  |
| S6132 | | Cortex lacn + open intracranial wound + <1hr loss of consc | |  |
| S6133 | | Cortex lacn + open intracranial wound + 1-24hr loss of consc | |  |
| S6134 | | Cortex lacn + open intracranial wound + >24hr LOC + recovery | |  |
| S6135 | | Cortex lacn + open intracranial wnd + >24hr LOC not restored | |  |
| S6136 | | Cortex lacn + open intracranial wnd + LOC unspec duration | |  |
| S613z | | Cortex lacn + open intracranial wound + concussion unspec | |  |
| S614. | | Closed hindbrain contusion | |  |
| S6140 | | Hind brain cont no open intracranial wnd +unspec state consc | |  |
| S6141 | | Hind brain cont no open intracranial wnd + no loss of consc | |  |
| S6142 | | Hind brain cont no open intracranial wnd +<1hr loss of consc | |  |
| S6143 | | Hind brain cont no open intracranial wnd + 1-24hr loss consc | |  |
| S6144 | | Hind brain cont no open intracranial wnd+>24hr LOC +recovery | |  |
| S6145 | | Hind brain cont open intracranial wnd+>24hr LOC not restored | |  |
| S6146 | | Hind brain cont no open intracranial wnd+LOC unspec duration | |  |
| S614z | | Hind brain cont no open intracranial wnd + concussion unspec | |  |
| S615. | | Open hindbrain contusion | |  |
| S6150 | | Hind brain cont + open intracranial wnd + unspec state consc | |  |
| S6151 | | Hind brain cont + open intracranial wound + no loss consc | |  |
| S6152 | | Hind brain cont + open intracranial wound + <1hr loss consc | |  |
| S6153 | | Hind brain cont + open intracranial wnd + 1-24hr loss consc | |  |
| S6154 | | Hind brain cont + open intracranial wnd +>24hr LOC +recovery | |  |
| S6155 | | Hind brain cont + open intracran wnd +>24hr LOC not restored | |  |
| S6156 | | Hind brain cont + open intracranial wnd +LOC unspec duration | |  |
| S615z | | Hind brain cont + open intracranial wnd + concussion unspec | |  |
| S616. | | Hind brain laceration without open intracranial wound | |  |
| S6160 | | Hind brain lacn no open intracranial wnd +unspec state consc | |  |
| S6161 | | Hind brain lacn no open intracranial wnd + no loss consc | |  |
| S6162 | | Hind brain lacn no open intracranial wnd + <1hr loss consc | |  |
| S6163 | | Hind brain lacn no open intracranial wnd + 1-24hr loss consc | |  |
| S6164 | | Hind brain lacn no open intracranial wnd +>24hr LOC+recovery | |  |
| S6165 | | Hind brain lacn no open intracran wnd+>24hr LOC not restored | |  |
| S6166 | | Hind brain lacn no open intracran wnd + LOC unspec duration | |  |
| S616z | | Hind brain lacn no open intracranial wnd + concussion unspec | |  |
| S617. | | Hind brain laceration with open intracranial wound | |  |
| S6170 | | Hind brain lacn + open intracranial wnd + unspec state consc | |  |
| S6171 | | Hind brain lacn + open intracranial wnd + no loss consc | |  |
| S6172 | | Hind brain lacn + open intracranial wnd + <1hr loss consc | |  |
| S6173 | | Hind brain lacn + open intracranial wnd + 1-24hr loss consc | |  |
| S6174 | | Hind brain lacn + open intracranial wnd+>24hr LOC + recovery | |  |
| S6175 | | Hind brain lacn open intracranial wnd+>24hr LOC not restored | |  |
| S6176 | | Hind brain lacn + open intracran wnd + LOC unspec duration | |  |
| S617z | | Hind brain lacn + open intracranial wnd + concussion unspec | |  |
| S61x. | | Oth cerebral laceration/contusion no open intracranial wound | |  |
| S61x0 | | Brain cont no open intracranial wound + unspec state consc | |  |
| S61x1 | | Brain cont no open intracranial wound + no loss of consc | |  |
| S61x2 | | Brain cont no open intracranial wound + <1hr loss of consc | |  |
| S61x3 | | Brain cont no open intracranial wound + 1-24hr loss of consc | |  |
| S61x4 | | Brain cont no open intracranial wound + >24hr LOC + recovery | |  |
| S61x5 | | Brain cont no open intracranial wnd + >24hr LOC not restored | |  |
| S61x6 | | Brain cont no open intracranial wound + LOC unspec duration | |  |
| S61xz | | Brain cont no open intracranial wound + concussion unspec | |  |
| S61y. | | Oth cerebral laceration/contusion + open intracranial wound | |  |
| S61y0 | | Brain cont + open intracranial wound + unspec state consc | |  |
| S61y1 | | Brain cont + open intracranial wound + no loss of consc | |  |
| S61y2 | | Brain cont + open intracranial wound + <1hr loss of consc | |  |
| S61y3 | | Brain cont + open intracranial wound + 1-24hr loss of consc | |  |
| S61y4 | | Brain cont + open intracranial wound + >24hr LOC + recovery | |  |
| S61y5 | | Brain contusion + open intracr wound+>24hr LOC not ful reco | |  |
| S61y6 | | Brain cont + open intracranial wound + LOC unspec duration | |  |
| S61yz | | Brain cont + open intracranial wound + concussion unspec | |  |
| S61z. | | Cerebral laceration and contusion NOS | |  |
| S62.. | | Cerebral haemorrhage following injury | |  |
| S620. | | Closed traumatic subarachnoid haemorrhage | |  |
| S6200 | | Subarachnoid h'ge inj no open intracran wound + unspec consc | |  |
| S6201 | | Subarachnoid h'ge inj no open intracran wnd+no loss consc | |  |
| S6202 | | Subarachnoid h'ge inj no open intracran wnd+<1hr loss consc | |  |
| S6203 | | Subarachnoid h'ge inj no open intracran wound + 1-24hr LOC | |  |
| S6204 | | Subarachnoid h'ge inj no open intracran wnd+>24 LOC+recovery | |  |
| S6205 | | Subarach h'ge inj no open intracran wnd+>24hrs LOC-restored | |  |
| S6206 | | Subarach h'ge inj no open intracran wnd+LOC unspec duration | |  |
| S620z | | Subarach h'ge inj no open intracran wnd + concussion unspec | |  |
| S621. | | Open traumatic subarachnoid haemorrhage | |  |
| S6210 | | Subarachnoid h'ge inj + open intracran wound + unspec consc | |  |
| S6211 | | Subarachnoid h'ge inj + open intracranial wound + no LOC | |  |
| S6212 | | Subarachnoid h'ge inj + open intracran wound+<1hr loss consc | |  |
| S6213 | | Subarachnoid h'ge inj + open intracran wnd+1-24hr loss consc | |  |
| S6214 | | Subarach h'ge inj + open intracran wnd +>24hr LOC + recovery | |  |
| S6215 | | Subarach h'ge inj + open intracran wnd+>24hr LOC -restored | |  |
| S6216 | | Subarach h'ge inj + open intracran wnd+LOC unspec duration | |  |
| S621z | | Subarachnoid h'ge inj + open intracran wnd+concussion unspec | |  |
| S622. | | Closed traumatic subdural haemorrhage | |  |
| S6220 | | Subdural h'ge inj no open intracranial wnd + unspec consc | |  |
| S6221 | | Subdural h'ge inj no open intracranial wound+no loss consc | |  |
| S6222 | | Subdural h'ge inj no open intracranial wound+<1hr loss consc | |  |
| S6223 | | Subdural h'ge inj no open intracran wnd+1-24hr loss consc | |  |
| S6224 | | Subdural h'ge inj no open intracranial wnd+>24 LOC +recovery | |  |
| S6225 | | Subdural h'ge inj no open intracran wnd+>24hr LOC -restored | |  |
| S6226 | | Subdural h'ge inj no open intracran wnd+LOC unspec duration | |  |
| S622z | | Subdural h'ge inj no open intracran wound+concussion unspec | |  |
| S623. | | Open traumatic subdural haemorrhage | |  |
| S6230 | | Subdural h'ge inj + open intracranial wound + unspec consc | |  |
| S6231 | | Subdural h'ge inj + open intracranial wound+no loss consc | |  |
| S6232 | | Subdural h'ge inj + open intracranial wound+<1hr loss consc | |  |
| S6233 | | Subdural h'ge inj + open intracranial wnd+1-24hr loss consc | |  |
| S6234 | | Subdural h'ge inj + open intracran wound+>24hr LOC +recovery | |  |
| S6235 | | Subdural h'ge inj + open intracran wnd+>24hr LOC -restored | |  |
| S6236 | | Subdural h'ge inj + open intracran wnd+LOC unspec duration | |  |
| S623z | | Subdural h'ge inj + open intracranial wnd+concussion unspec | |  |
| S624. | | Closed traumatic extradural haemorrhage | |  |
| S6240 | | Extradural h'ge inj no open intracranial wnd + unspec consc | |  |
| S6241 | | Extradural h'ge inj no open intracranial wnd + no loss consc | |  |
| S6242 | | Extradural h'ge inj no open intracranial wnd+<1hr loss consc | |  |
| S6243 | | Extradural h'ge inj no open intracran wnd+1-24hr loss consc | |  |
| S6244 | | Extradural h'ge inj no open intracran wnd+>24hr LOC+recovery | |  |
| S6245 | | Extradural h'ge inj no open intracran wnd+>24hr LOC-restored | |  |
| S6246 | | Extradural h'ge inj no open intracra wnd+LOC unspec duration | |  |
| S624z | | Extradural h'ge inj no open intracran wnd+concussion unspec | |  |
| S625. | | Open traumatic extradural haemorrhage | |  |
| S6250 | | Extradural h'ge inj + open intracranial wnd + unspec consc | |  |
| S6251 | | Extradural h'ge inj + open intracranial wound+no loss consc | |  |
| S6252 | | Extradural h'ge inj + open intracranial wnd+<1hr loss consc | |  |
| S6253 | | Extradural h'ge inj + open intracran wnd+1-24hr loss consc | |  |
| S6254 | | Extradural h'ge inj + open intracran wnd+>24hr LOC+recovery | |  |
| S6255 | | Extradural h'ge inj + open intracran wnd+>24hr LOC -restored | |  |
| S6256 | | Extradural h'ge inj + open intracran wnd+LOC unspec duration | |  |
| S625z | | Extradural h'ge inj + open intracran wnd+concussion unspec | |  |
| S626. | | Epidural haemorrhage | |  |
| S627. | | Traumatic subarachnoid haemorrhage | |  |
| S628. | | Traumatic subdural haemorrhage | |  |
| S629. | | Traumatic subdural haematoma | |  |
| S6290 | | Traumatic subdural haematoma without open intracranial wound | |  |
| S6291 | | Traumatic subdural haematoma with open intracranial wound | |  |
| S62A. | | Traumatic extradural haematoma | |  |
| S62A0 | | Traumatic extradural haemat without open intracranial wound | |  |
| S62A1 | | Traumatic extradural haematoma with open intracranial wound | |  |
| S62z. | | Cerebral haemorrhage following injury NOS | |  |
| S63.. | | Other cerebral haemorrhage following injury | |  |
| S630. | | Other cerebral h'ge after injury no open intracranial wound | |  |
| S6300 | | Oth cerebral h'ge inj no open intracran wnd+unspec consc | |  |
| S6301 | | Oth cerebral h'ge inj no open intracranial wnd+no loss consc | |  |
| S6302 | | Oth cerebral h'ge inj no open intracran wnd+<1hr loss consc | |  |
| S6303 | | Oth cerebral h'ge inj no open intracran wnd+1-24hr LOC | |  |
| S6304 | | Oth cereb h'ge inj no open intracran wnd+>24hr LOC +recovery | |  |
| S6305 | | Oth cereb h'ge inj no open intracran wnd+>24hr LOC -restored | |  |
| S6306 | | Oth cereb h'ge inj no open intracran wnd+LOC unspec duration | |  |
| S630z | | Oth cereb h'ge inj no open intracran wnd+concussion unspec | |  |
| S631. | | Other cerebral h'ge after injury + open intracranial wound | |  |
| S6310 | | Oth cerebral h'ge inj + open intracran wnd + unspec consc | |  |
| S6311 | | Oth cerebral h'ge inj + open intracranial wnd+no loss consc | |  |
| S6312 | | Oth cerebral h'ge inj + open intracran wnd+<1hr loss consc | |  |
| S6313 | | Oth cerebral h'ge inj + open intracran wnd+1-24hr loss consc | |  |
| S6314 | | Oth cereb h'ge inj + open intracran wnd+>24hr LOC + recovery | |  |
| S6315 | | Oth cereb h'ge inj + open intracran wnd+>24hr LOC -restored | |  |
| S6316 | | Oth cereb h'ge inj + open intracran wnd+LOC unspec duration | |  |
| S631z | | Oth cereb h'ge inj + open intracran wnd+concussion unspec | |  |
| S63z. | | Other cerebral haemorrhage following injury NOS | |  |
| S64.. | | Intracranial injury NOS | |  |
| S640. | | Intracranial injury NOS no open intracranial wound | |  |
| S6400 | | Intracranial inj NOS no open intracran wnd + unspec consc | |  |
| S6401 | | Intracranial inj NOS no open intracran wnd+no loss consc | |  |
| S6402 | | Intracranial inj NOS no open intracran wnd+<1hr loss consc | |  |
| S6403 | | Intracranial inj NOS no open intracran wnd+1-24hr loss consc | |  |
| S6404 | | Intracranial inj NOS no open intracran wnd+>24hr LOC+recover | |  |
| S6405 | | Intracran inj NOS no open intracran wnd+>24hr LOC -restored | |  |
| S6406 | | Intracran inj NOS no open intracran wnd+LOC unspec duration | |  |
| S640z | | Intracranial inj NOS no open intracran wnd+concussion unspec | |  |
| S641. | | Intracranial injury NOS + open intracranial wound | |  |
| S6410 | | Intracranial inj NOS + open intracranial wnd + unspec consc | |  |
| S6411 | | Intracranial inj NOS + open intracranial wound+no loss consc | |  |
| S6412 | | Intracranial inj NOS + open intracranial wnd+<1hr loss consc | |  |
| S6413 | | Intracranial inj NOS + open intracranial wnd + 1-24hr LOC | |  |
| S6414 | | Intracranial inj NOS + open intracran wnd+>24hr LOC+recovery | |  |
| S6415 | | Intracran inj NOS + open intracran wnd+>24hr LOC -restored | |  |
| S6416 | | Intracran inj NOS + open intracran wnd+LOC unspec duration | |  |
| S641z | | Intracranial inj NOS + open intracran wnd+concussion unspec | |  |
| S642. | | Traumatic cerebral oedema | |  |
| S6420 | | Traumatic cerebral oedema without open intracranial wound | |  |
| S6421 | | Traumatic cerebral oedema with open intracranial wound | |  |
| S643. | | Diffuse brain injury | |  |
| S644. | | Focal brain injury | |  |
| S645. | | Intracranial injury with prolonged coma | |  |
| S6450 | | Intracranial injury with prolonged coma without open wound | |  |
| S6451 | | Intracranial injury with prolonged coma with open wound | |  |
| S646. | | Head injury | |  |
| S6460 | | Minor head injury | |  |
| S64z. | | Intracranial injury NOS | |  |
| S6z.. | | Intracranial injury, excluding those with skull fracture NOS | |  |
| S83.. | | Other open wound of head | |  |
| S830. | | Open wound of scalp | |  |
| S8301 | | Avulsion of scalp | |  |
| S831. | | Open wound of scalp with complication | |  |
| S83x. | | Other open wound of head | |  |
| S83y. | | Other open wound of head with complication | |  |
| S83z. | | Open wound of head NOS | |  |
| S8W.. | | Injury of blood vessels of head, NEC | |  |
| S8X.. | | Traumatic amputation of unspecified part of head | |  |
| SD0.. | | Superficial injury to head, excluding eye | |  |
| SE09. | | Contusion, scalp | |  |
| SF01. | | Crush injury, scalp | |  |
| SF03. | | Crushing injury of skull | |  |
| SF0X. | | Crushing injury of head, part unspecified | |  |
| SJ0.. | | Optic nerve and pathway injury | |  |
| SJ00. | | Optic nerve (2nd) injury | |  |
| SJ01. | | Optic chiasm injury | |  |
| SJ02. | | Optic pathway injury | |  |
| SJ03. | | Visual cortex injury | |  |
| SJ0z. | | Optic nerve or pathway injury NOS | |  |
| SJ1.. | | Injury to other cranial nerves | |  |
| SJ10. | | Oculomotor (3rd) nerve injury | |  |
| SJ11. | | Trochlear (4th) nerve injury | |  |
| SJ12. | | Trigeminal (5th) nerve injury | |  |
| SJ13. | | Abducens (6th) nerve injury | |  |
| SK1x0 | | Multiple injuries of head | |  |
| Syu0. | | [X]Injuries to the head | |  |
| Syu01 | | [X]Superficial injury of other parts of head | |  |
| Syu02 | | [X]Open wound of other parts of head | |  |
| Syu03 | | [X]Fractures of other skull and facial bones | |  |
| Syu04 | | [X]Fracture of skull and facial bones, part unspecified | |  |
| Syu05 | | [X]Dislocation of other and unspecified parts of head | |  |
| Syu06 | | [X]Sprain/strain of joint/ligam of oth & unsp part of head | |  |
| Syu07 | | [X]Injury of other cranial nerves | |  |
| Syu0D | | [X]Other intracranial injuries | |  |
| Syu0E | | [X]Intracranial injury, unspecified | |  |
| Syu0F | | [X]Crushing injury of other parts of head | |  |
| Syu0G | | [X]Crushing injury of head, part unspecified | |  |
| Syu0H | | [X]Traumatic amputation of other parts of head | |  |
| Syu0J | | [X]Traumatic amputation of unspecified part of head | |  |
| Syu0K | | [X]Injury of blood vessels of head, NEC | |  |
| Syu0L | | [X]Other specified injuries of head | |  |
| **VITAMIN B-12 DEFCIENCY** | | | |  |
| D0111 | | Vit.B12 defic.anaemia-malabs. | |  |
| D0121 | | Folate-defic. anaemia-dietary | |  |
| D0123 | | Folate-defic. anaemia – malabs | |  |
| 1452 | | H/O: Anaemia vit.B12 deficient | |  |
| D011X | | Vit B12 defic anaemia, unsp | |  |
| D011. | | Other vit.B12 defic. Anaemias | |  |
| D0124 | | Folate-defic.anaemia-liver dis | |  |
| D0110 | | Vit.B12 defic.anaemia-dietary | |  |
| D012. | | Folate-deficiency anaemia | |  |
| D0120 | | Congen.folate malabs.anaemia | |  |
| D012z | | Folate-deficiency anaemia NOS | |  |
| Dyu02 | | [X]Other vit B12 defic anaemia | |  |
| Dyu06 | | [X]Vit B12 defic anaemia, unsp | |  |
| **ANAEMIA** | | | |  |
| D01y. | | Other nutrit. defic. anaemia | |  |
| 1454 | | H/O: anaemia NOS | |  |
| 2C2.. | | O/E – anaemia | |  |
| D001. | | Iron defic.anaemia-dietary | |  |
| D21yy | | Other anaemia OS | |  |
| D21y. | | Other specified anaemias | |  |
| D00y. | | Other spec. iron defic.anaemia | |  |
| D00zz | | Iron deficiency anaemia NOS | |  |
| D01yy | | Other nutrit.defic.anaemia OS | |  |
| 2C22. | | O/E - equivocally anaemic | |  |
| 2C24. | | O/E - profoundly anaemic | |  |
| D01.. | | Other deficiency anaemias | |  |
| D0z.. | | Deficiency anaemias NOS | |  |
| D014. | | Protein-deficiency anaemia | |  |
| 2C2Z. | | O/E - anaemia NOS | |  |
| Dyu0. | | [X]Nutritional anaemias | |  |
| D010. | | Pernicious anaemia | |  |
| D214. | | Chronic anaemia | |  |
| 2C23. | | O/E - clinically anaemic | |  |
| D000. | | Iron defic.anaemia-blood loss | |  |
| Dyu00 | | [X]Oth iron deficncy anaemias | |  |
| Dyu22 | | [X]Anaemia in oth chron dis CE | |  |
| 1451 | | H/O: anaemia - iron deficient | |  |
| D01yz | | Other nutrit.defic.anaemia NOS | |  |
| D01z. | | Other deficiency anaemias NOS | |  |
| D00z. | | Unspec iron deficiency anaemia | |  |
| D21.. | | Other and unspecified anaemias | |  |
| D2z.. | | Other anaemias NOS | |  |
| D0... | | Deficiency anaemias | |  |
| D00.. | | Iron deficiency anaemias | |  |
| D0y.. | | Deficiency anaemias OS | |  |
| D2012 | | Aplastic anaemia-infection | |  |
| D21yz | | Other specified anaemia NOS | |  |
| D21z. | | Anaemia unspecified | |  |
| Dyu24 | | [X]Other specified anaemias | |  |
| **ANXIOLYTICS** | | | |  |
| d2% | | Anxiolytics | |  |
| **ANTIDEPRESSANTS** | | | |  |
| d6% | | Lithium salts | |  |
| d7% | | Tricyclic antidepressants | |  |
| d8% | | Monoamine oxidase inhibitors | |  |
| d9% | | Compound anti-depressant drugs | |  |
| da% | | Other anti-depressant drugs | |  |
| **ANTICOAGULANTS** | | | |  |
| bs% | | Oral anticoagulants | |  |
| **ANTIPLATELETS** | | | | |
| bu% | | Antiplatelet drugs | | |
| **STATINS** | | | | |
| bxe1. | | *PRAVASTATIN 10mg tablets | | |
| bxe2. | | *PRAVASTATIN 20mg tablets | | |
| bxi.. | | ATORVASTATIN | | |
| bxi1. | | ATORVASTATIN 10mg tablets | | |
| bxi2. | | ATORVASTATIN 20mg tablets | | |
| bxi3. | | ATORVASTATIN 40mg tablets | | |
| bxiz. | | ATORVASTATIN 80mg tablets | | |
| bxg3. | | FLUVASTATIN 20mg capsules | | |
| bxg4. | | FLUVASTATIN 40mg capsules | | |
| bxgz. | | FLUVASTATIN 80mg m/r tablets | | |
| bxg.. | | FLUVASTATIN SODIUM | | |
| bxe.. | | PRAVASTATIN SODIUM | | |
| bxe5. | | PRAVASTATIN SODIUM 10mg tabs | | |
| bxe6. | | PRAVASTATIN SODIUM 20mg tabs | | |
| bxe7. | | PRAVASTATIN SODIUM 40mg tabs | | |
| bxk.. | | ROSUVASTATIN | | |
| bxkx. | | ROSUVASTATIN 10mg tablets | | |
| bxky. | | ROSUVASTATIN 20mg tablets | | |
| bxkz. | | ROSUVASTATIN 40mg tablets | | |
| bxkw. | | ROSUVASTATIN 5mg tablets | | |
| bxd.. | | SIMVASTATIN | | |
| bxd1. | | SIMVASTATIN 10mg tablets | | |
| bxd2. | | SIMVASTATIN 20mg tablets | | |
| bxdu. | | SIMVASTATIN 20mg/5mL oral susp | | |
| bxdy. | | SIMVAST 20mg/EZETIMIB 10mg tab | | |
| bxd5. | | SIMVASTATIN 40mg tablets | | |
| bxdv. | | SIMVASTATIN 40mg/5mL oral susp | | |
| bxdx. | | SIMVAST 40mg/EZETIMIB 10mg tab | | |
| bxdz. | | SIMVASTATIN 80mg tablets | | |
| bxdw. | | SIMVAST 80mg/EZETIMIB 10mg tab | | |
| **HORMONE REPLACEMENT THERAPY** | | |  |  |
| ff% | | Oestrogens |  |  |
| fg% | | Progesterones |  |  |
| fh% | | Combined osterogen + progesterone preperations |  |  |
| **VITAMIN B-12 SUPPLEMENTATION** | | |  |  |
| in% | | Vitamin B group |  |  |
| **ANAEMIA MEDICATION** | | |  |  |
| i1% | | Iron deficiency – oral iron |  |  |

**S3 Table: Univariate/candidate variables to be included in model**

|  | **Hazard ratio** | **Difference (95% CI)** |
| --- | --- | --- |
| PPI | 0.82 | 0.80 to 0.84* |
| Age | 1.07 | 1.07 to 1.08 |
| Female | 1.26 | 1.24 to 1.29* |
| BMI | 0.93 | 0.93 to 0.93* |
| Drink alcohol | 1.20 | 1.13 to 1.26* |
| Smoker | 0.91 | 0.89 to 0.94* |
| Diabetes | 0.95 | 0.92 to 0.97* |
| Cardiovascular disease | 1.04 | 1.02 to 1.06* |
| Depression | 1.65 | 1.62 to 1.69* |
| Head injury | 1.78 | 1.72 to 1.85* |
| Vitamin B_12_ Deficiency | 1.76 | 1.60 to 1.94* |
| Anaemia | 1.26 | 1.23 to 1.29* |
| Anxiolytics | 2.03 | 1.99 to 2.08* |
| Antidepressants | 1.95 | 1.91 to 1.99* |
| Anticoagulants | 0.82 | 0.79 to 0.84* |
| Antiplatelets | 1.66 | 1.62 to 1.70* |
| Statins | 0.78 | 0.76 to 0.79* |
| Hormone replacement therapy | 0.77 | 0.74 to 0.80* |
| Vitamin B_12_ Medication | 1.86 | 1.78 to 1.93* |
| Anaemia Medication | 1.36 | 1.33 to 1.39* |
| Antihypertensives | 0.79 | 0.77 to 0.81* |
| Histamine Receptor- 2 Medication | 0.95 | 0.93 to 0.97* |

**S4 Table: Hazard ratio of developing dementia when taking a PPI compared to a control population using a retrospective cohort while omitting vitamin-B_12_ deficiency for sensitivity analysis.**

|  | **Hazard ratio** | **95% CI** |
| --- | --- | --- |
| PPI | 0.66 | 0.64 to 0.69* |
| Age | 1.06 | 1.05 to 1.06* |
| Female | 0.97 | 0.94 to 1.00 |
| Drink alcohol | 1.17 | 1.08 to 1.26* |
| Smoker | 0.89 | 0.86 to 0.92* |
| BMI | 0.95 | 0.95 to 0.95* |
| Diabetes | 1.18 | 1.15 to 1.23* |
| Cardiovascular disease | 0.93 | 0.90 to 0.96* |
| Depression | 1.20 | 1.16 to 1.24* |
| Head injury | 1.50 | 1.43 to 1.57* |
| Anaemia | 1.04 | 1.00 to 1.08* |
| Anxiolytics | 1.63 | 1.58 to 1.68* |
| Antidepressants | 1.67 | 1.61 to 1.72* |
| Anticoagulants | 0.87 | 0.84 to 0.91* |
| Antiplatelets | 1.73 | 1.67 to 1.80* |
| Statins | 0.85 | 0.82 to 0.88* |
| Hormone replacement therapy | 0.89 | 0.85 to 0.94* |
| Anaemia Medication | 1.11 | 1.07 to 1.14* |
| Antihypertensives | 0.71 | 0.69 to 0.73* |
| Histamine Receptor- 2 Medication | 0.92 | 0.88 to 0.96* |

*p=<0.01*
